# Differences in Extracellular Vesicle Protein Cargo Are Dependent on Head and Neck Squamous Cell Carcinoma Cell of Origin and Human Papillomavirus Status

**DOI:** 10.3390/cancers13153714

**Published:** 2021-07-23

**Authors:** Christine Goudsmit, Felipe da Veiga Leprevost, Venkatesha Basrur, Lila Peters, Alexey Nesvizhskii, Heather Walline

**Affiliations:** 1 Department of Otolaryngology-Head and Neck Surgery, University of Michigan, Ann Arbor, MI 48109, USA; cgoud@umich.edu (C.G.); peters@umich.edu (L.P.); 2 Department of Pathology, University of Michigan, Ann Arbor, MI 48109, USA; felipevl@umich.edu (F.d.V.L.); vbasrur@umich.edu (V.B.); nesvi@umich.edu (A.N.); 3 Proteomics Shared Resource, Department of Pathology, University of Michigan, Ann Arbor, MI 48109, USA

**Keywords:** HPV, head and neck squamous cell carcinoma, extracellular vesicle, proteomic

## Abstract

**Simple Summary:**

Many individuals with head and neck cancer do not survive, even with intense treatment. Patients with HPV-positive tumors generally have better survival; however, for yet unknown reasons, a subset are unresponsive to therapy. One strategy to monitor cancers for progression and recurrence is evaluation of extracellular vesicles, released by tumor cells into the blood and other body fluids. We can also understand differences in tumors and their behavior by comparing the molecules packaged into vesicles that are released from tumor cells. Our study examined differences in the proteins contained within extracellular vesicles released from head and neck cancer cells. We found that key extracellular vesicle proteins differed based on HPV status of the originating cell line and tumor, as well as how responsive the originating tumor was to treatment. Our findings suggest that these extracellular vesicle proteins may be important markers for continued investigation.

**Abstract:**

To identify potential extracellular vesicle (EV) biomarkers in head and neck squamous cell carcinoma (HNSCC), we evaluated EV protein cargo and whole cell lysates (WCL) from HPV-positive and -negative HNSCC cell lines, as well as normal oral keratinocytes and HPV16-transformed cells. EVs were isolated from serum-depleted, conditioned cell culture media by polyethylene glycol (PEG) precipitation/ultracentrifugation. EV and WCL preparations were analyzed by LC-MS/MS. Candidate proteins detected at significantly higher levels in EV compared with WCL, or compared with EV from normal oral keratinocytes, were identified and confirmed by Wes Simple Western protein analysis. Our findings suggest that these proteins may be potential HNSCC EV markers as proteins that may be (1) selectively included in EV cargo for export from the cell as a strategy for metastasis, tumor cell survival, or modification of tumor microenvironment, or (2) representative of originating cell composition, which may be developed for diagnostic or prognostic use in clinical liquid biopsy applications. This work demonstrates that our method can be used to reliably detect EV proteins from HNSCC, normal keratinocyte, and transformed cell lines. Furthermore, this work has identified HNSCC EV protein candidates for continued evaluation, specifically tenascin-C, HLA-A, E-cadherin, EGFR, EPHA2, and cytokeratin 19.

## 1. Introduction

Head and neck squamous cell carcinoma (HNSCC) is the sixth most common and eighth most fatal cancer worldwide and includes cancers of the oropharynx, larynx, hypopharynx, and oral cavity [1,2]. In 2018, there were 890,000 new cases and 450,000 deaths worldwide, and annual cases are expected to reach 1.08 million by 2030 [3]. Despite recent advances in treatment, including radiation, chemotherapy, surgery, concurrent chemoradiation, and immunotherapy, many tumors develop resistance and progress. Patients develop metastases or tumors recur locally or regionally; the 5-year overall survival rate for HNSCC is only 40–50% [4]. Furthermore, the majority of patients suffer at least one recurrence, typically within 2 years of treatment [5,6,7]. Factors that contribute to poor survival for patients with HNSCC include late stage diagnosis, lack of reliable markers for early stage detection, high level of biologic heterogeneity, and local recurrence and distant metastases after treatment [3].

There are subsets of HNSCC that respond better to therapy; 70–80% of HPV-positive oropharyngeal squamous cell carcinomas (OPSCCs) treated with concurrent chemotherapy and intensity-modulated radiation or surgery with lymph node dissection and post-operative radiation respond favorably. This improved outcome suggests that some patients could be treated with reduced-intensity treatments, sparing them the severe morbidities associated with current therapies [8,9]. However, de-escalation efforts risk increasing the current 20% of HPV-positive OPSCCs that are non-responsive to treatment. Unfortunately, regardless of treatment, many patients have tumors that progress or recur, and we are unable to distinguish those that are likely to respond to reduced treatment from tumors that will require additional or alternate therapies.

Extracellular vesicles (EVs) are membrane-enclosed particles released from a cell (constitutively, upon activation, or under hypoxic conditions), that contain various biological molecules, including signaling factors, proteins, nucleic acids, and lipids. The International Society of Extracellular Vesicles (ISEV) defines EVs as “particles released from a cell that are delimited by a lipid bilayer and cannot replicate (i.e., lack a functional nucleus)” [10]. These vesicles carry biologically active cytosolic and membrane components of the parent cell, allowing them to serve as originator surrogates [11]. They differ in size, density, function, and content, and include exosomes, microvesicles, and large oncosomes. Exosomes are small (40–150 nm) membrane-bound vesicles with a characteristic cup-shaped morphology that originate in the endosomes of cells, resulting in formation of multivesicular bodies, which are subsequently released into the extracellular space through fusion with the plasma membrane of the cell. Microvesicles and oncosomes are generated by outward budding from the plasma membrane of non-apoptotic cells, and are significantly larger than exosomes; microvesicles range in size from 0.1–10 µm, and oncosomes are 1–10 µm.

EVs are important because they are produced by both normal and tumor cells and have many functions, including roles in the immune response, inflammation, intercellular messaging, and transport [12,13]. Tumor extracellular vesicles (TEVs) are taken up by recipient cells locally in the primary tumor and tumor microenvironment (fibroblasts, endothelial cells, immune cells, or other tumor cells) as well as in distant cells and tissues, resulting in delivery of tumor molecules that can alter recipient cells [14,15,16]. Tumor cells are prolific vesicle producers, and TEVs have the capability to mediate pro-tumorigenic effects through immunomodulation, angiogenesis, promotion of tumor growth, modulation of the tumor microenvironment, and transport of oncogenic signals or active oncogenes from tumor cells to normal cells [11,13,17,18]. Extracellular vesicles affect recipient cells by docking to the surface to transmit signals or transferring their contents into the cell.

Proteins within EVs are protected from degradation in biological fluids, allowing for increased stability of these molecules for liquid biopsy evaluation compared with circulating tumor cells or cell-free circulating tumor molecules [19]. Some cancer-specific proteins released from HNSCC cells in culture have been previously identified, as well as proteins present in the saliva or circulation of HNSCC patients [20,21]. The goals of this study were to identify additional EV protein markers that may be (1) selectively included in EV cargo for export from the cell as a strategy for metastasis, tumor cell survival, or modification of tumor microenvironment, or (2) representative of originating cell composition, which may be developed for diagnostic or prognostic use in clinical liquid biopsy applications.

## 2. Materials and Methods

Cell culture: This study used 8 representative HNSCC cell lines (Table 1), one HPV-transformed cell line, HOK16b (Human Oral Keratinocytes-16A, RRID: CVCL_B404) [22], and two non-cancer oral keratinocytes, NOKsi (normal oral keratinocytes, spontaneously immortalized) [23] and HOKg (human oral keratinocytes from gingiva, Lifeline Cell Technologies Oceanside, CA), using methods established and optimized in our lab that adhere to the 2018 MISEV guidelines. The total number of cell lines used in the mass spec analysis [10] was chosen so that the samples could be assayed concurrently to reduce variation. These specific cell lines were chosen to include multiple HNSCC tumor sites (oral cavity, hypopharynx, larynx, and oropharynx) and HPV status. The non-responsive HNSCC cell lines (UM-SCC-38, UM-SCC-47, UM-SCC-118, UM-SCC-104, and UPCI:SCC152) were generated from more aggressive tumors that were resistant to treatment. These tumors were removed from patients with disease ultimately causal in their deaths. The responsive HNSCC cell lines (UM-SCC-17a, UM-SCC-105, and UM-SCC-92) were generated from tumors removed from patients with tumors responsive to treatment, and at last query the patients were alive with no evidence of disease.

All cell lines were grown in T-150 flasks in 20 mL standard media containing serum. Upon reaching approximately 80% confluence, the media was removed, cells were rinsed twice with PBS, and exosome-depleted media (EDM) was added (Exosome-depleted Fetal Bovine Serum, Thermo Fisher #A2720801). Following 48 h of incubation, the conditioned cell culture media was collected for EV isolation. Three T-150 flasks were used for each HNSCC cell line, and 9 T-150 flasks were used for HOK16b and NOKsi cells.

Contamination testing and genetic assessment was performed on all cell lines prior to freezing, upon thawing, and monthly during the course of cell culture experiments. Cell lines were tested for mycoplasma contamination using the Lonza MycoAlert Mycoplasma Detection Kit and genotyped in the University of Michigan Genomics Core using ProfilerPlus, which interrogates 15 tetranucleotide short tandem repeats (STR), to confirm unique genotypes. The cell lines were also tested for known genetic characteristics including key mutations (using exon sequencing) and HPV status/type (using the HPV PCR-MassArray assay).

EV isolation by ultracentrifugation: Our EV ultracentrifugation method was modified from the isolation strategy described by Thery, et. al. [24]. Briefly, conditioned cell culture media was combined from replicate flasks, with each set including a 5 mL PBS wash (Corning #21-040-CV, without Calcium or Magnesium). Samples were precleared by centrifugation at 300× *g* for 10 min in 50 mL conical tubes, using an Eppendorf centrifuge (Model #5810R) at 4 °C. The supernatants were transferred to new tubes and centrifuged at 2800× *g* for 10 min with the same parameters as above. Supernatants were then transferred to ultracentrifuge tubes (OptiSeal Polypropylene Bell Top Tubes, Beckman Coulter #361625), and were centrifuged at 12,000× *g* for 30 min at 4 °C (SW 28 Swinging-Bucket Aluminum Rotor, Beckman Coulter #342204/Beckman Optima XL-100K Ultracentrifuge). Supernatants were transferred to new tubes (Quick-Seal Polypropylene Tubes, Beckman Coulter #342413) and centrifuged at 110,000× *g* for 90 min at 4 °C (Type 70 Ti Fixed-Angle Titanium Rotor, Beckman Coulter #337922/Beckman Optima XL-100K Ultracentrifuge). The supernatants were removed, and the pellets were resuspended in 1 mL PBS and transferred to fresh tubes. The original pellet tubes were washed twice with 1 mL PBS each time, and this was added to the resuspended pellets in the new tubes, for a combined total of 3 mL. The volume was brought to 13 mL with additional PBS before final centrifugation at 110,000× *g* for 90 min at 4 °C (Type 70 Ti rotor). Supernatants were removed and pellets were stored at −80 °C.

EV isolation by PEG precipitation/ultracentrifugation: This protocol was based on the PEG method from Rider et al. [25]. EVs were isolated by polyethylene glycol (PEG) precipitation followed by ultracentrifugation. Briefly, conditioned cell culture media was removed from the flasks, with each washed with 5 mL PBS and transferred to a 50 mL conical tube. Samples were precleared by centrifugation at 300× *g* for 10 min in 50 mL conical tubes using an Eppendorf centrifuge (Model #5810R) at 4 °C. Supernatants were transferred to new tubes and centrifuged at 2800× *g* for 10 min with the same parameters as above. Supernatants were then transferred to new 50 mL conical tubes and centrifuged at 10,000× *g* for 30 min at 4 °C (Sorvall ST 8R centrifuge with HIGHConic Fixed-Angle rotor).

Supernatants were combined with an equal volume of 16% PEG and 1 M NaCl, for a final concentration of 8% PEG, 0.5 M NaCl, and incubated overnight at 4 °C. The PEG precipitations were then pelleted at 3220× *g* for 60 min, using an Eppendorf centrifuge at 4 °C. The supernatants were removed and discarded, leaving approximately 2 mL on each of the pellets. The pellets were resuspended and transferred to ultracentrifuge tubes (Quick-Seal Polypropylene Tubes, Beckman Coulter #342413); the precipitation tubes were each washed twice with PBS and the washes were added to the pellet suspensions. The samples were each brought up to 13 mL total with PBS and centrifuged at 150,000× *g* for 120 min at 4 °C (Type 70 Ti rotor). Supernatants were removed and pellets were stored at −80 °C.

Electron microscopy: EVs were pelleted as described above and resuspended in 80 µL PBS. An amount of 10 µL of the resuspended pellet was fixed with an equal volume of 5% glutaraldehyde in 0.2 M cacodylate buffer, pH 7.2. After (10 µL/15 min) application to formvar/carbon coated EM grids (200 mesh copper), they were washed with dH_2_O (3 × 1 min). Grids were then incubated for 5 min in 1% uranyl acetate for negative contrasting. Images were taken using a Jeol JEM-1400 Plus 120 keV transmission electron microscope equipped with C-MOS camera and AMT (Advanced Microscopy Technology) software (ver. 6.02).

Nanoparticle Tracking Analysis (NTA): EV size and concentration for each sample isolation was determined using a NanoSight NS300 instrument (NanoSight, Malvern, UK) using a Blue405 laser and sCMOS camera. Samples were diluted in PBS, and 100 µL aliquots were measured with three 60 s videos using a syringe pump speed of 100 at a controlled temperature of 25 °C. Videos were analyzed with NTA 3.2 Build 3.2.16 software to calculate particle size and concentration for each sample.

Protein extraction: EVs isolated by ultracentrifugation and associated whole cell lysates were prepared using 1% NP40 in PBS for mass spectrometry. EVs isolated by PEG precipitation/ultracentrifugation and associated whole cell lysates were prepared with Roche Complete Mini Protease Inhibitor Cocktail Tablets (catalog # 11836153001) for Western analysis, and quantified with the Bradford protein assay.

Mass spectrometry: Proteomic analysis was performed on EVs and whole cell lysates from the eight HNSCC cell lines, normal NOKsi cell line, and the transformed cell line, HOK16b. Relative quantitation of proteins using Tandem Mass Tag (TMT 10plex) and LC-Tandem MS was performed by the Proteomics Resource Facility at the Department of Pathology, University of Michigan, using an optimized protocol described by Tank et al. [26]. Briefly, equal amounts of each protein sample were digested, labeled with TMT 10plex reagents, and subjected to Multinotch MS3 method as previously described [27]. MS data analysis was performed using Proteome discoverer 2.1 (ThermoFisher (Waltham, MA, USA). Protein identification and relative quantification was achieved by searching the data against a Homo sapiens UniProtKB database (UP000005640) that was downloaded on 21 September 2018. Protein sequences from HPV16 and HPV18 were also added to the FASTA file bringing the total number of proteins to 73136. EV and whole cell lysate protein results were compared to identify differences between normal and carcinoma EVs and whole cell lysates, as well as between EVs from HPV-positive and –negative HNSCC cell lines. 

Wes ProteinSimple Western: Protein was measured using a capillary-based electrophoresis instrument (Wes, ProteinSimple, San Jose, CA, USA). Protein amounts (0.25–1 µg) pre-optimized for specific antibodies were denatured using manufacturer-supplied reagents and loaded into multi-well plates. Protein separation and detection were performed via capillary electrophoresis, antibody binding, and HRP-conjugated visualization following the manufacturer’s instructions. Thorough antibody optimization was completed for all proteins for which Wes analysis was performed to determine ideal input protein concentration/denaturation, specific antibody (supplier/clone), and dilution conditions. Antibodies used are listed in Table 2. Analysis was performed using the Compass software for Simple Western (ProteinSimple, San Jose, CA, USA).

## 3. Results

### 3.1. Extracellular Vesicle Characterization

**Figure 1:**
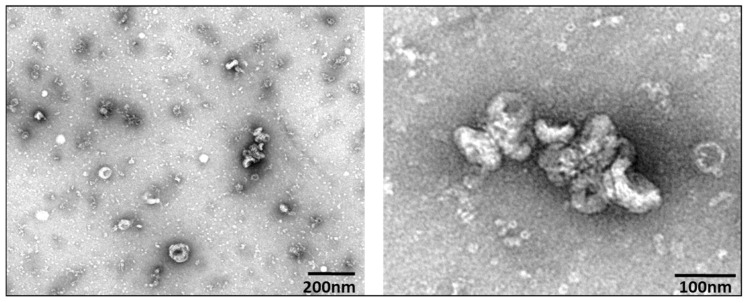
Representative TEM, UM-SCC-104, showing EVs 75–100 nm in diameter, with characteristic cup-shaped morphology.

**Figure 2:**
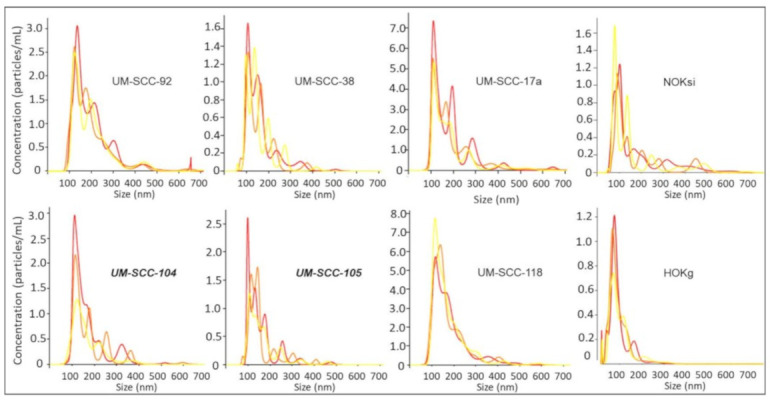
Nanoparticle tracking analysis, showing size and concentration for EVs from six representative HNSCC cell lines and two non-cancer cell lines measured. Bold italics: HPV-positive.

EV characterization was based on minimal information for studies of extracellular vesicles 2018 [1]. Representative TEM images are shown in Figure 1, revealing EVs that demonstrate classic EV cup-shape morphology and diameter range between 75 and 200 nm. NTA measurements at 10-fold and 100-fold dilutions indicated that EVs isolated from cell culture supernatants yielded an average particle size of 177 nm across the HNSCC lines measured (Figure 2), while the average concentration was 9.22 × 10^9^ particles/mL.

### 3.2. Western Confirmation of EV Markers

**Figure 3:**
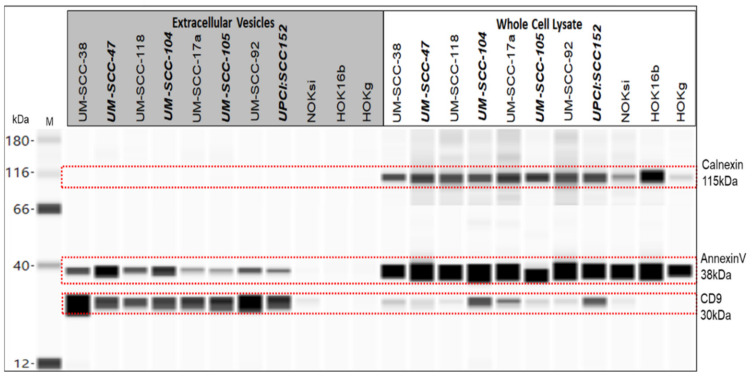
Wes protein analysis. Calnexin 1:50, 1 µg/µL protein; annexinV 1:200, 0.25 µg/µL protein; CD9 1:25, 1 µg/µL protein. Bold italics: HPV-positive.

(Figure 3) Positive EV marker CD9 was detected at 30 kDa in all of the EV lysates tested (HOK16b and HOKg bands are faint but present, see peak chemiluminescence, Figure S1, panel A). Similarly, AnnexinV, another positive EV marker, was detected at 38 kDa in all of the EVs tested (peak chemiluminescence shown in Figure S1, panel B). Calnexin, considered a negative marker for small EVs [10,28,29], was detected at 115 kDa in all of the whole cell lysates tested and none of the EV samples tested.

### 3.3. Proteomic Analysis by Mass Spectrometry

**Figure 4:**
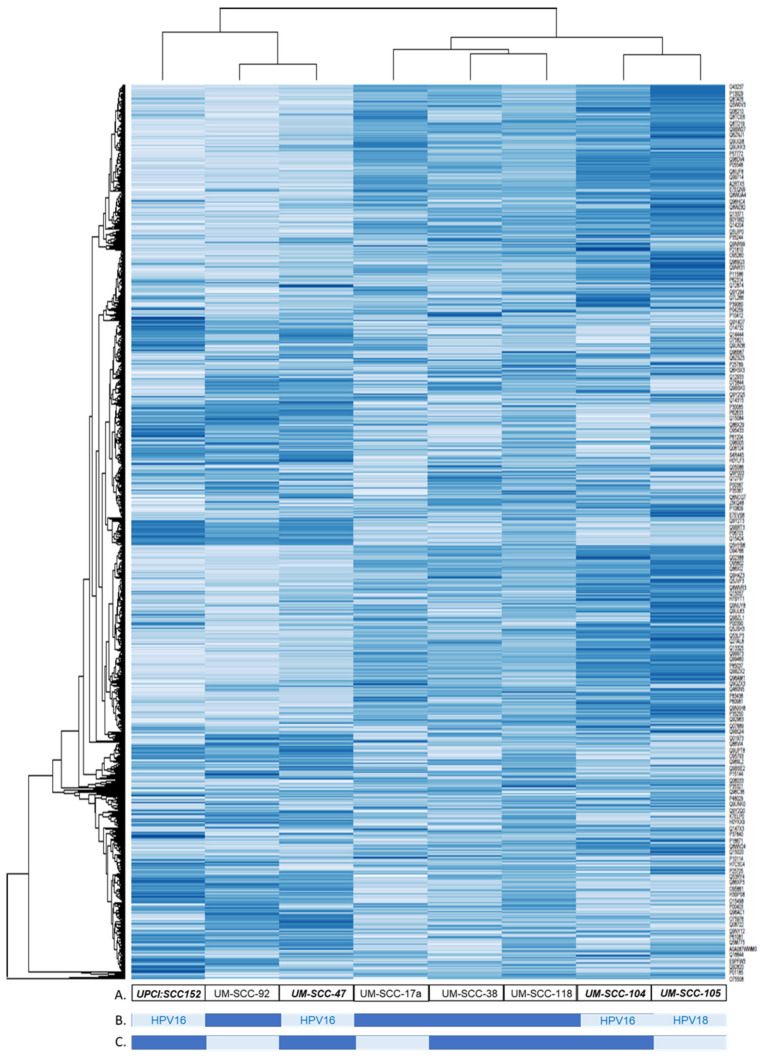
Cluster analysis of fold-change HNSCC EV protein abundance compared with NOKsi EV. protein. (**A**) UMSCC cell line EV identifier. (**B**) HPV status; light = HPV-positive, dark = HPV-negative. (**C**) Responsive/non-responsive origin tumor; light = responsive, dark = non-responsive. Bold italics: HPV-positive.

**Figure 5:**
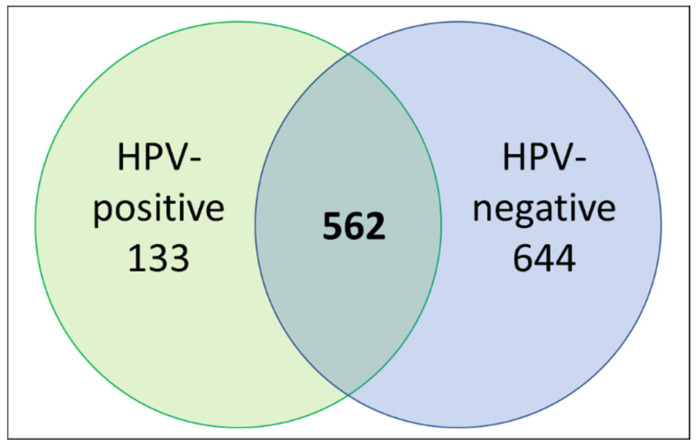
Venn diagram illustrating the distribution of proteins in EVs released from HPV-positive and HPV-negative HNSCC cell lines by averaged PSM values.

The coverage—calculated by dividing the number of amino acids in all found peptides by the total number of amino acids in the entire protein sequence—of proteins were also taken into consideration for selection of potential protein markers, with higher coverage values considered to be more likely to yield promising candidates. A total of 4945 proteins were identified from the analysis of extracellular vesicles, and 5738 proteins were identified from the analysis of whole cell lysates. HNSCC EV fold-change peptide spectrum matches (PSM) for the EVs from the HNSCC lines compared with the EVs from the normal keratinocyte line (NOKsi) were averaged together. There were 149 proteins identified that were >2; these are represented in the cluster analysis (Figure 4) and listed in Table S1. Proteins in EVs released from HPV-positive and -negative HNSCC cell lines were further compared to determine proteins common to both groups or exclusive to EVs from either HPV-positive or -negative lines (Figure 5). EVs from HPV-negative HNSCC cell lines exclusively contained 644 proteins; 133 proteins were detected exclusively in those from HPV-positive lines, and 562 were common in EVs from both HPV-positive and -negative HNSCC cell lines.

### 3.4. Western Analysis of Candidate Proteins

Candidate EV proteins were initially considered based on the proteomic analysis by mass spectrometry (using the coverage and comparative PSM values), and final candidates were selected by either previously reported involvement in tumors at multiple HNSCC sites, relationship with HPV tumorigenic pathways, or likelihood of EV association, including membrane proteins.

Protein results by ProteinSimple Wes analysis were not normalized to an internal control protein. Limitations inherent to working with EVs, recognized by the ISEV, prevent absolute normalization of expression data, due to variations in composition of EVs from different cell lines [30]. The ISEV recommends combining high-resolution imaging of isolated EVs with concentration measurements, as was performed in our study.

**Figure 6:**
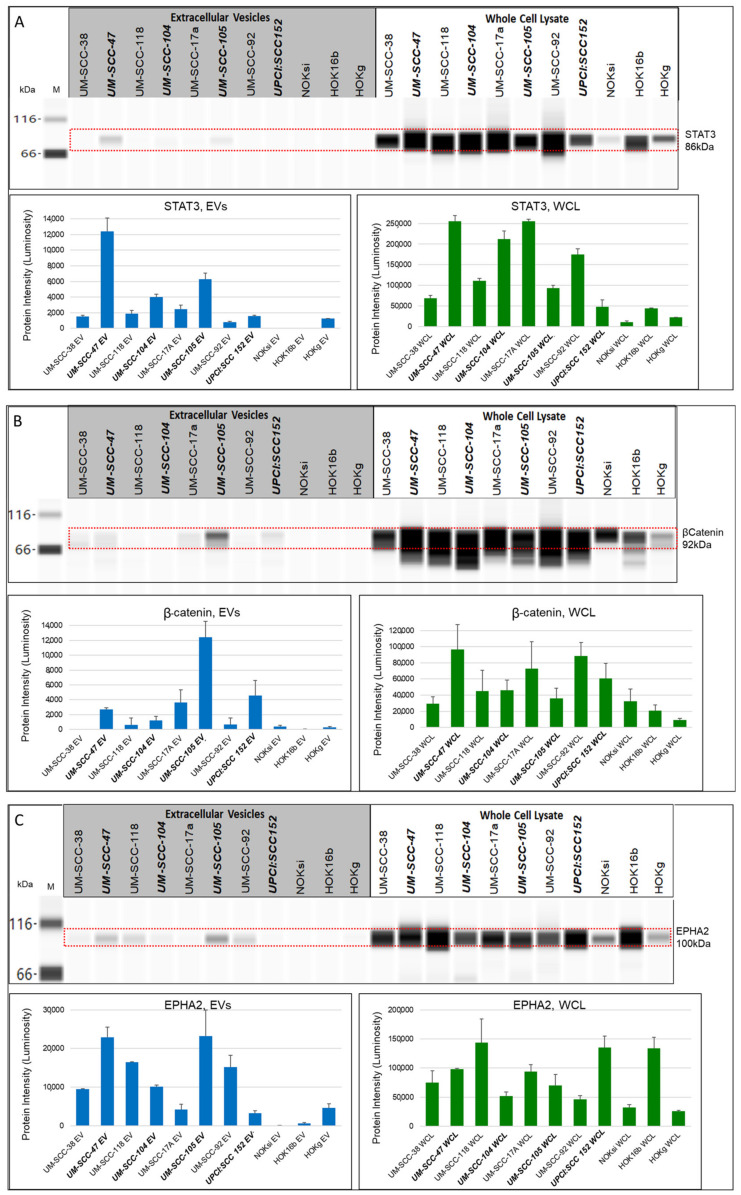

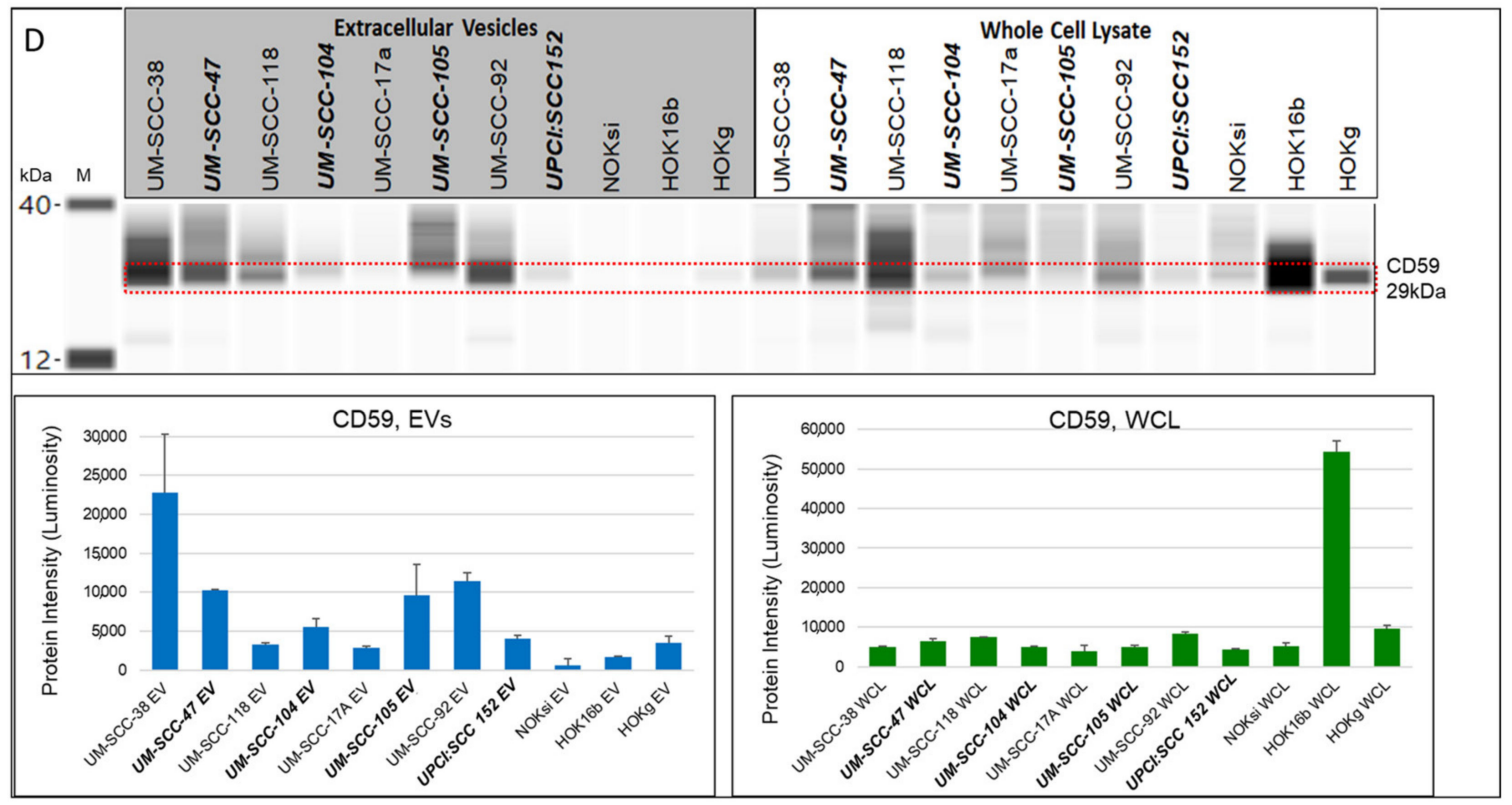
Wes protein gel and quantitative luminosity for extracellular vesicles and whole cell lysates from HNSCC, normal keratinocyte, and transformed cell lines. (**A**) STAT3 detected at 86 kDa, 1:25 antibody dilution, 0.5 μg/μL protein; (**B**) βCatenin detected at 92 kDa, 1:200 antibody dilution, 0.25 μg/μL protein; (**C**) EPHA2 detected at 100 kDa,1:100 antibody dilution, 0.5 mg/mL protein; and (**D**) CD59 detected at 29 kDa,1:100 antibody dilution, 1:7, 1 mg/mL protein. Bold italics: HPV-positive.

STAT3 (Figure 6A) was detected at 86 kDa in all of the whole cell lysates, with a much fainter band in the whole cell lysate from the normal NOKsi cell line. Overall, the STAT3 levels were generally detected at higher levels in EVs from the HPV-positive HNSCC lines compared with those from the HPV-negative lines. This was not the case for the whole cell lysates, where HPV-negative lines UM-SCC17A, UM-SCC-92, and UM-SCC-118 all had relatively high levels of STAT3 detected. 

βCatenin (βCat) (Figure 6B) was detected at 92 kDa in all of the whole cell lysate samples tested. In the EVs, the only appreciable βCatenin signals were seen in the EVs from HPV-positive HNSCC lines UM-SCC-47, UM-SCC-105, and UPCI:SCC152, and those from the HPV-negative HNSCC line UM-SCC-17A.

EPHA2 (Figure 6C) was detected with a strong signal at 100 kDa in all of the whole cell lysates tested. The protein was detected at varying levels in EVs from all the cell lines tested with the exception of those from NOKsi, where no EPHA2 protein was detected.

CD59 (Figure 6D) was detected at 29 kDa in the whole cell lysates of all the lines tested, with the highest levels detected in the transformed cell line, HOK16a. This protein was detected in the EVs from all HNSCC lines, with the highest level seen in HPV-negative UM-SCC-38. 

**Figure 7:**
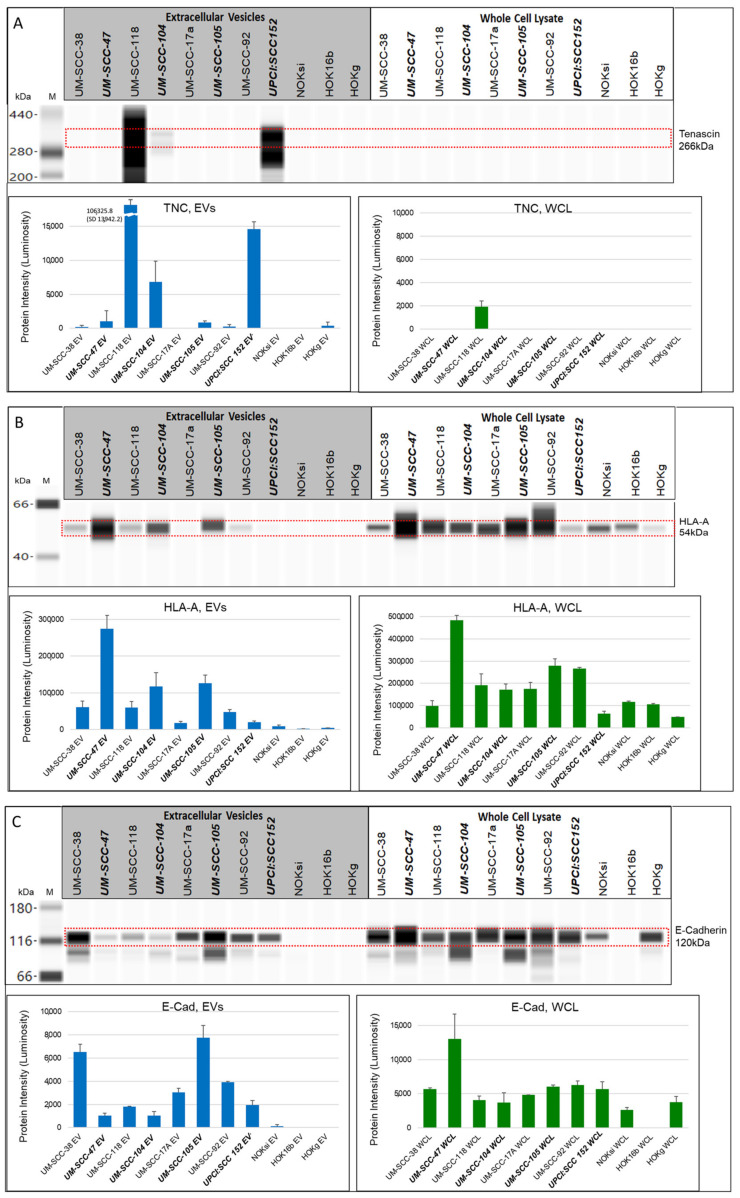

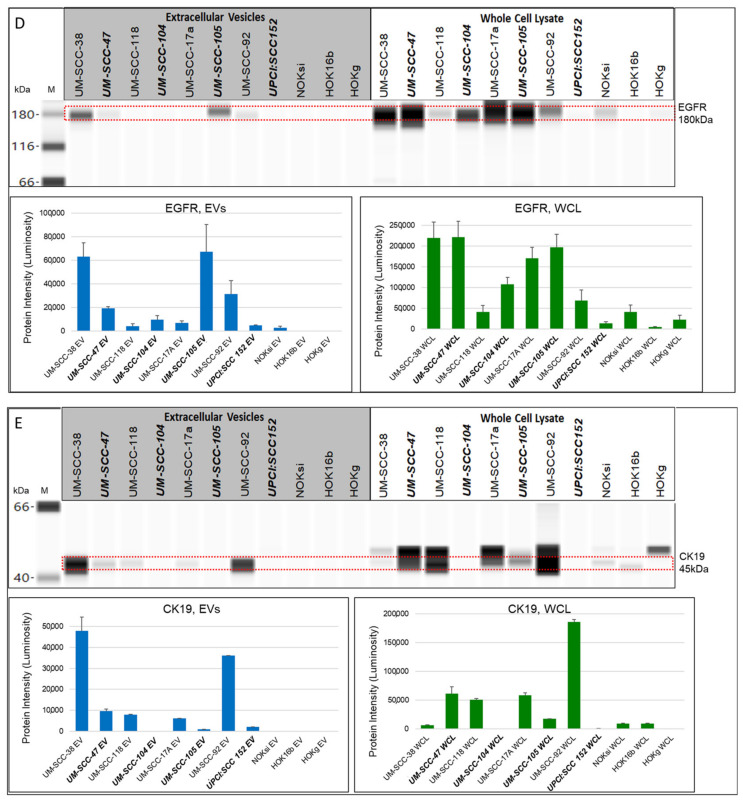
Wes protein gel and quantitative luminosity for extracellular vesicles and whole cell lysates from HNSCC, normal keratinocyte, and transformed cell lines. (**A**) Tenascin detected at 266 kDa, 1:200 antibody dilution, 0.5 μg/μL protein; (**B**) HLA-A detected at 54 kDa, 1:400 antibody dilution, 0.25μg/μL protein; (**C**) E-cadherin detected at 120 kDa, 1:250 antibody dilution, 0.5μg/μL protein; (**D**) EGFR detected at 180 kDa, 1:100 antibody dilution, 0.25 μg/μL protein; and (**E**) CK19 detected at 45 kDa, 1:100 antibody dilution, 0.25 mg/mL protein. Bold italics: HPV-positive.

Despite tenascin-C (TNC) (Figure 7A) being detected in the whole cell lysate of only one (UM-SCC-118A) of the eleven cell lines tested, it was seen in several lines across the EV panel. Tenascin-C was detected at 266 kDa in EVs from the HPV-positive HNSCC lines, UM-SCC-47, UM-SCC-104, UM-SCC-105, and UPCI:SCC152, and at incredibly high levels in EVs from the HPV-negative cell line UM-SCC-118a. Fainter bands were detected in EVs from HPV-negative UM-SCC-38 and UM-SCC-92, and those from normal cell line HOKg.

HLA-A (Figure 7B) was detected at 54 kDa in all of the whole cell lysates with the highest intensity band in the whole cell lysate from the HPV-positive HNSCC cell line, UM-SCC-47. In the EVs, HLA-A was detected at the highest levels in EVs from the HPV-positive HNSCC lines, UM-SCC-47, UM-SCC-104, and UM-SCC-105. HLA-A was not detected in EVs from NOKsi or HOKg lines.

E-cadherin (E-Cad) (Figure 7C) was detected at 120 kDa in all of the whole cell lysates tested with the exception of the HPV transformed line, HOK16B. E-cadherin was also detected in all of the HNSCC EVs tested. Interestingly, the EVs from HPV-positive cell lines UM-SCC-47, UM-SCC-104, and UPCI:SCC152 (those generated from non-responsive tumors) had lower levels of E-cadherin detected than EVs from HPV-negative UM-SCC-17, or UM-SCC-92, or EVs from HPV18-positive UM-SCC-105 (generated from a responsive tumor).

EGFR (Figure 7D) was detected in all of the whole cell lysates tested. EGFR was detected in all of the HNSCC EVs. Two of the EV samples with the highest levels of EGFR were those from HPV18-positive UM-SCC-105 and HPV-negative UM-SCC-92; these cell lines were derived from tumors that were responsive to treatment and the patients are still alive. 

Cytokeratin 19 (CK19) (Figure 7E) was detected at the highest levels in EV from HPV-negative cell lines UM-SCC-38 and UM-SCC-92, while the protein is also seen in the whole cell lysates of the HPV-negative cell lines as well as HPV-positive UM-SCC-47 and UM-SCC-105. 

**Figure 8:**
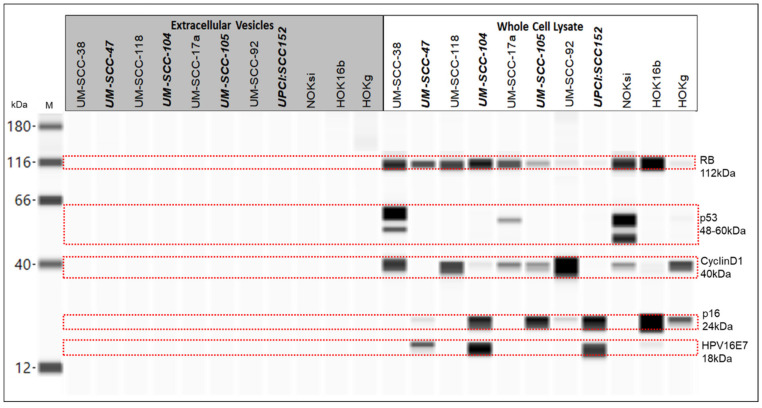
Wes protein gel for extracellular vesicles and whole cell lysates from HNSCC, normal keratinocyte, and transformed cell lines. RB detected at 112 kDa, 1:100 antibody dilution, 0.5 mg/mL protein; p53 detected at 60 kDa, 52 kda, and 48 kDa, 1:50 antibody dilution, 0.25 μg/μL protein; CyclinD1 detected at 40 kDa, 1:200 antibody dilution, 0.25 μg/μL protein; p16 detected at 24 kDa, no antibody dilution, 0.25 μg/μL protein; and HPV16E7 detected at 18 kDa, 1:100 antibody dilution, 1 μg/μL protein. Bold italics: HPV-positive.

HPV16 and associated proteins, RB, p53, CyclinD1, and p16 are shown in Figure 8. HPV16E7 was detected at 18 kDa in only the whole cell lysates of the HPV16-positive cell lines tested, UM-SCC-47, UM-SCC-104, UPCI:SCC152, and the HPV16-transformed cell line, HOK16b. HPV16E7 was not detected in any of the EVs tested. 

The 112 kDa form of RB protein was detected in all of the whole cell lysates, and none of the EVs, from all of the eleven cell lines tested. The protein was detected at varying levels in the different whole cell lysates. 

p53 was detected in only whole cell lysates from HPV-negative cell lines UM-SCC-38 (at 52 kDa and 60 kDa) and UM-SCC-17a (at 60 kDa), as well as whole cell lysates from the normal cell line NOKsi (at 48 kDa and 60 kDa). There was also a faint signal in the normal HOKg whole cell lysate sample at 60 kDa. p53 was not detected in any of the EVs tested.

CyclinD1 was detected at 40 kDa in all of the whole cell lysates tested with the exceptions of HPV-positive HNSCC cell lines UM-SCC-47 and UPCI:SCC152, where no CyclinD1 protein was found. There was no CyclinD1 protein detected in any of the EVs tested.

None of the EVs tested contained detectable levels of p16 protein. We did identify p16 at 24 kDa in whole cell lysates of HPV-positive HNSCC cell lines UM-SCC-47, UM-SCC-104, UM-SCC-105, and UPCI:SCC152, as well as HPV-negative HNSCC line UM-SCC-92. The whole cell lysates of the transformed cell line HOK16b and normal HOKg cell line also contained p16 protein, although the lysate of the normal NOKsi line did not.

## 4. Discussion

**Figure 9:**
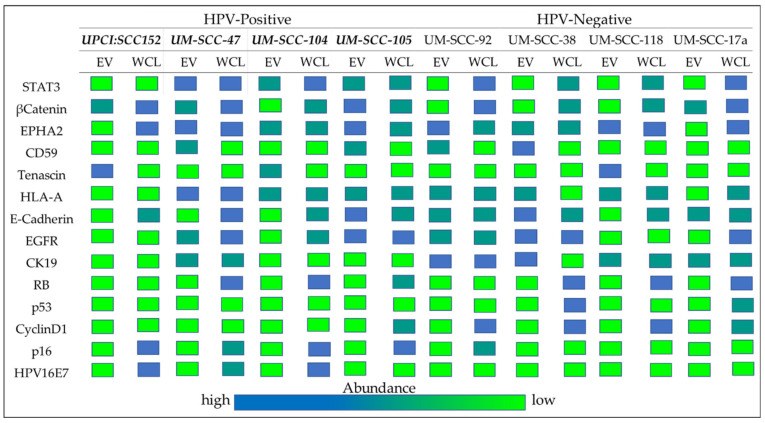
Relative abundance of the selected proteins detected by Wes protein analysis in EVs and WCL from HNSCC cell lines, by HPV status. Bold italics: HPV-positive.

The proteins detected in the EVs from the HNSCC cell lines were expected to recapitulate the characteristics of the originating cells. This study suggests that this is frequently not the case, as seen in Figure 9, which illustrates the comparative protein levels detected in WCL and EV from HNSCC cell lines queried in this study. While the EV protein cargo does not consistently represent that of the parent cell, there are striking differences between EV proteins that could be important indicators of tumor cell behavior and these warrant further investigation. Furthermore, the proteins detected in EVs from tumor cells are likely purposely intended for transport from the cell as a mechanism for tumor cell survival, growth, or other advantage.

Tenascin-C is an extracellular matrix glycoprotein, expressed during embryogenesis, organogenesis, wound healing and inflammation [31,32,33,34]. TNC has been well studied, and has many known binding sites for cell surface receptors (including integrins) and extracellular matrix proteins (including fibronectin); multiple integrins and fibronectin were candidates in our proteomics analysis [31,32,33,34,35].

Tenascin-C has been implicated in many cancer types: esophageal, breast, colorectal, bladder, Merkel cell carcinomas, glioma, malignant pleural mesothelioma, glioblastoma multiforme, malignant melanoma, and astrocytoma, and has been implicated in a variety of roles: tumorigenesis, angiogenesis, migration, proliferation, maintaining the stemness of cancer stem cells, and metastasis [31,32,36,37,38,39,40,41]. TCGA data have shown increased mRNA expression in HNSCC [37]. TNC has also been shown to suppress T cell activation [37,42]. TNC is implicated in HPV-positive cervical cancer, although its mechanism is still unknown [33,43]. TNC-containing exosomes were found in association with glioblastoma patients having a T cell-suppressive role [37]. Our Wes protein results showed TNC in two subsets: EVs from HNSCC cell line UM-SCC-118, generated from an HPV-negative tongue cancer, and EVs from all four HPV-positive HNSCC cell lines (UM-SCC-47, UM-SCC-104, UM-SCC-105, and UPCI:SCC152). TNC is seen more often in stroma cells than tumor cells, so it is not surprising that TNC was not detected in the majority of WCL, whether HPV-positive or -negative [44]. It is interesting that TNC expression was seen in UM-SCC-118 WCL, an HPV-negative cell line, and that the protein was detected in the EVs as well. The detection of TNC in HPV-positive EVs establishes the possibility of TNC as a marker for HPV-positive HNSCC. It has been studied previously as a biomarker for HNSCC (unrelated to HPV), with mixed results [35,40,45,46], while others have investigated TNC (associated with HPV) and found TNC to be involved but do not know the exact mechanism of action [33]. It is of particular interest that TNC was seen in such distinct EV groups, and makes this protein a candidate for further investigation.

The signal transducer and activator of transcription 3 (STAT3) is part of the signal transducer and activator of the transcription protein family. STAT3 plays a pivotal role in tumorigenesis through transcription regulation of genes having essential roles in cell proliferation, inflammation, differentiation, apoptosis, angiogenesis, immune response, and metastasis [47]. STAT3 has many known receptors including the Janus kinases, EGFR, and IL-6 [47,48,49]. In this study, STAT3 was detected in the EVs from all the HPV-positive HNSCC cell lines. The highest expression was in the EVs from UM-SCC-47, followed by those from UM-SCC-105, the only HPV18 positive line. Recent studies have shown that HPV18 causes activation of STAT3 through the HPV18 E6 protein [50,51]. There are also general implications that STAT3 is activated by viruses, which would account for the high levels of STAT3 in all of the HPV-positive whole cell lysates [52]. Based on these results, STAT3 would be a reasonable EV protein candidate for HPV-positive HNSCC, if it had not also been detected in the EVs from the non-cancer HOKg cell line, making this protein a less desirable candidate for progression in our studies.

HLA (human leukocyte antigen) is part of the MHC class I immune system. There are classical forms, HLA-A, HLA-B, and HLA-C, and non-classical forms, HLA-G, HLA-E, HLA-F, and HLA-H, of this protein; both have been identified in cancer with diverse roles [53,54,55,56,57]. In our study, we identified HLA-A in WCL from all of the lines tested and in EVs from all of the HNSCC lines. HLA-A was detected in the EVs from three of the HPV-positive HNSCC lines at higher levels than in EVs from the other HNSCC lines; this might support the use of HLA-A and allelotyping for prognosis in HPV-positive HNSCC, which is currently used in cervical carcinomas and to a lesser extent in HNSCC [53,58,59,60,61,62,63,64,65,66]. It would be worthwhile to look at these methods from the EV perspective to provide a less invasive predictive or prognostic biomarker. HLA-E has previously been suggested to offer a predictive prognosis in laryngeal lesions [53]. Although not tested by Wes protein analysis, many of the non-classical HLA forms were present in our EV MS proteomics data, with the exception of HLA-G. These non-classical HLA proteins participate in immune system evasion [54,56,57]. A recent study showed that the use of monalizumab blocked the binding of NKG2A in the presence of HLA-E, causing an increase in both natural killer and T cells. When used in combination with cetuximab, the authors showed a 31% objective response rate [67]. This study, taken together with our results, demonstrates that HLA proteins are robust EV protein candidates for continued investigation. 

β-Catenin, together with the cadherin family of adhesion proteins, is part of the Wnt signaling pathway, and is thought to have roles in both HPV-positive and -negative HNSCC and be associated to de-differentiation and poor prognosis [68,69,70,71]. HPV-16 oncoprotein regulates the translocation of β-catenin via the activation of epidermal growth factor receptor. β-catenin is normally seen on cell membrane, but when it is cleaved from E-cadherin and not phosphorylated for degradation, it accumulates in the cytoplasm and is translocated to the nucleus by the T cell factor (TCF)/lymphoid enhancer-binding factor (LEF) transcriptional factors, ultimately leading to epithelial–mesenchymal transition (EMT) [71,72,73,74]. β-catenin has downstream targets associated with the tumor microenvironment including extracellular matrix components, laminin, and fibronectin; this relationship with the microenvironment may be supported through molecular transport by EVs [71]. The highest EV signal for β-catenin was seen in the EVs from HPV18-positive HNSCC UM-SCC-105, and was also detected in EVs from two other HPV-positive HNSCC lines. 

E-cadherin is part of the cadherin family of proteins, and functions as a main adhesion protein of the epithelia with key roles in attachment with the formation of E-cadherin/β-catenin complex, polarity, and structure. Repression, downregulation, or loss of E-cadherin is correlated with metastasis and invasion and has been implicated in EMT [73,75,76,77,78,79,80,81,82]. E-cadherin is associated with a number of pathways, including Wnt/β-catenin, ERK, MAP kinase, and PI3K-Akt [77,83]. In HNSCC, reduced E-cadherin expression has been shown to be associated with poor prognosis, tumor aggressiveness, EMT completion, metastasis, and lower overall survival [80,81,82,84,85]. Historically, EPHA2 has been evaluated together with E-cadherin, as it is dependent on E-cadherin for localization to cell–cell sites. In the absence of E-cadherin, EPHA2 does not transfer to the cell–cell site and cells exhibit pro-metastatic behavior [86]. In HNSCC, loss of E-cadherin is characteristic of advanced tumor stage and metastatic potential [80,81,82,84,85]. In our study, E-cadherin was detected in the EVs from all of the HNSCC lines, but not the EVs from the normal keratinocyte lines. Furthermore, the E-cadherin levels detected in EVs from HPV-16 positive HNSCC lines UM-SCC-47, UM-SCC-104, and UPCI:SCC152 were overall lower than those in EVs from HPV-negative or HPV18-positive UM-SCC-17A, UM-SCC-105, and UM-SCC-92. This finding makes E-cadherin an especially attractive EV protein candidate for further investigation.

EphA2 is a receptor tyrosine kinase that binds to adjacent cells through binding to surface-associated Ephrin (Eph receptor interacting protein) ligands [87]. Dysfunction/dysregulation within the Eph family of proteins has been seen in several types of disease, ranging from cancer to inflammation and a variety of cell types with sometimes contradictory implications owning to its bidirectional and promiscuous signaling [87,88]. Eph proteins have been implicated in cellular adhesion, angiogenesis, cell migration and proliferation, survival, differentiation, and secretion [87,88,89]. Based on its involvement in carcinogenesis and other pathologies, EphA2 has been evaluated as a target for drug therapy [90,91,92,93]. In HNSCC, EphA2 has been further investigated for its role in inflammation, epithelial–mesenchymal transition, migration, proliferation, and point of viral entry [94,95,96,97]. The HNSCC TCGA subset showed EphA2 to be among the significantly mutated genes [98]. More recently, it has been suggested as a potential compensatory mechanism in MET inhibition [99]. In our study, EphA2 was detected in all of the whole cell lysates tested and all of the EVs tested with the exception of the EVs from one of the normal keratinocyte lines (NOKsi). Overexpressed EPHA2 is shown to increase resistance to common EGFR drug therapy, cetuximab, via EPHA2 mediated activation of the PI3K/AKT pathway [86]. Overexpression of EPHA2 is also seen in cells resistant to erlotinib, gefitinib, and afatinib. [100,101]. EPHA2 together with EGFR may be a feasible marker combination to predict anti-EGFR resistance, making both of these proteins attractive candidates for further investigation.

EGFR is a type I receptor tyrosine kinase with a structure that spans the cell membrane and includes an extracellular ligand-binding domain and an intracellular kinase domain. EGFR is expressed in most epithelial tissues, and is involved in multiple pathways, including PI3K/Akt/mTOR, JAK/STAT, and RAS/RAF/MEK/ERK [81,102,103]. Many of these pathways involve mutations that become carcinogenic drivers [104]. EGFR has been implicated in many other cancer types, including breast, cervical, astrocytoma, bladder, esophageal, gastric, lung adenocarcinoma, colorectal, ovarian, and others [105,106,107,108,109,110,111,112,113]. EGFR is frequently overexpressed in HNSCC and a variety of other cancers [114]. In HNSCC, EGFR plays roles in invasion, migration, survival, proliferation, and metastasis [81,102,115]. While EGFR is expressed in HPV-positive and -negative cell lines (as seen in whole cell lysates in our study as well), there has been an association shown between EGFR and high-risk HPV E5 proteins in HPV-positive tumors, with HPV E5 having a role in activating EGFR [116,117,118,119]. EGFR has been studied as a candidate for targeted drug therapy in HNSCC; however, while overexpression is seen in 80–90% of HNSCC, only 10–20% are responsive to anti-EGFR drug therapy [102,115,120,121]. In our data, we see the same overexpression of EGFR in the Wes results with WCL and EVs, but with an overall lower level of overexpression in the EVs when compared with WCL levels. In other studies, EGFR is linked to EPHA2, and EPHA2 signaling is suggested to contribute to anti-EGFR therapy resistance [86].

Cytokeratin 19 is part of the cellular cytoskeletal network of intermediate filaments (IF), in the sub-classification of type I epithelial keratins [122,123]. Keratins have a widely accepted role in protection and structure of the cell, but recently they have been investigated for involvement in different cancers [122,123,124,125,126,127,128,129]. CK19 plays a major role in wound healing and tissue remodeling and has a variety of responses to the stresses (both mechanical and non-mechanical) endured by cells, ranging from cell signaling, migration, cell growth, differentiation, post translational modification, protein regulation, transcriptional regulation, and even metastasis, but the mechanism of action is largely yet unknown [122,123,124,126,130,131,132,133,134]. Keratins are considered a marker for epithelial stem cells, and increased cytokeratin 19 expression has been shown to be a biomarker of highly invasive oral squamous cell carcinoma with metastatic potential, as well as higher tumor recurrence and lower survival [129,130,135,136,137,138]. The role of CK19 in HNSCC in liquid biopsies (and thus our interest in evaluating this protein in EVs) was prompted through the use of keratin proteins for immunohistochemical tumor diagnosis in carcinomas. In particular, CK5, CK7, CK8, CK18, CK19, and CK20 [122]. The majority of these were included in our proteomic analysis, and of these CK19 had the most robust PSM and coverage. In addition to CK19, several other keratin proteins were identified in our proteomic study, including CK17, CK14, CK8, CK18, and CK6A. The raw proteomic data suggest a possible pattern of keratins present in the EVs from a subset of cell lines. Furthermore, a group in Japan is using CK19 as a marker for HNSCC in liquid biopsies [139]. This is supported by our results, showing no cytokeratin 19 detected in any of the non-cancer EVs. Another interesting finding from our Wes results is the relatively low abundance of CK19 in the UM-SCC-38 whole cell lysate, while the protein is detected at the highest levels in EVs from that same cell line. This protein and other cytokeratin family members will be further investigated as possible EV proteins released from tumor cells to participate in modification of the tumor microenvironment or similar mechanisms for survival and metastasis.

CD59 is a cell surface inhibitor of the membrane attack complex present in most tissues and on all circulating cells [140,141]. It is overexpressed on tumor cells, enabling tumor cells to escape from complement-mediated killing, listed in the top 50 proteins in the human proteomic atlas as an unfavorable prognostic protein [140,141,142,143]. CD59 has been evaluated as a salivary biomarker for oral squamous cell carcinomas together with M2BP, S100A9/MRP14, and catalase, achieving diagnostic sensitivity of 90%, specificity of 83%, and AUROCC of 0.93 for OSCC detection [144,145]. CD59 has also been shown to be present in exosomes [146], consistent with our findings. Unfortunately, our study also detected CD59 by Wes in the EVs from the non-cancer HOKg cell line, making this protein a less desirable candidate for progression in our studies.

HPV and associated proteins, RB, p53, Cyclin D1, and p16, are common biomarkers for HPV-positive and -negative HNSCC. HPV16E7 is a viral oncoprotein expressed in the HPV16-positive HNSCC tumors and cell lines. We thought that it might be detected in the EVs from the HPV16-positive cell lines; however, it was identified only in the whole cell lysates, including the whole cell lysate of the HPV16-transformed cell line, HOK16b. Similarly, we did not detect HPV-associated proteins RB, p53, Cyclin D1, or p16 in any of the EVs tested. We will evaluate other HPV proteins (including HPV18E6 and E7, as appropriate, and HPV16E6) as potential EV markers, or perhaps investigate alternate antibodies, as HPV proteins and several of the other HPV-associated markers have previously been identified in EVs from HPV-positive cell lines [147]. It was somewhat surprising that HPV proteins (E6 and E7) were not detected by MS in EVs from the tested HPV-positive cell lines. Another group detected HPV proteins in exosomes from one of the same HPV-positive cell lines we tested, UM-SCC-47 [147]. This is not entirely unfounded, as the isolation methods used to obtain EVs, the type of vesicles extracted/evaluated, and the method of sample preparation used for MS were different between the studies. 

## 5. Conclusions

We have demonstrated that proteins can be reliably detected in EVs and whole cell lysates from HNSCC, normal keratinocytes, and transformed cell lines, and that EVs released by HNSCC cells contain different proteins based on characteristics of the originating cell, including HPV status. Our work has importantly demonstrated that EVs released by tumor cells do not necessarily recapitulate the complete protein profile of the originating cell, and hypothesize that EV protein cargo is determined by other factors that are advantageous for the tumor cell, such as growth, immune evasion, modification of the tumor microenvironment, cell-to-cell communication, drug resistance, etc. Finally, this work has identified several protein candidates for continued evaluation for HNSCC EV markers, including tenascin-C, HLA-A, E-cadherin, EGFR, EPHA2, and cytokeratin 19. 

## Figures and Tables

**Table 1:** Head and Neck Squamous Cell Carcinoma Lines Used in the Study. Bold italics: HPV-positive.

Cell Line	Tumor Site	Tumor Stage	HPV Status	Patient Sex	Patient Age	Smoker?	Follow-Up
UM-SCC-38	Tonsil	T2N2aM0	Negative	Male	60	Yes	Patient died from disease one year after diagnosis
***UM-SCC-47***	Lateral Tongue	T3N1M0	HPV16	Male	53	Yes	Patient died from disease within a year of diagnosis
UM-SCC-118	Lateral Tongue	T4aN2M0	Negative	Female	23	No	Patient died from disease within a year of diagnosis
***UM-SCC-104***	Floor of Mouth	T4N2bM0	HPV16	Male	56	Yes	Patient died from disease within a year of diagnosis
UM-SCC-17a	Larynx	T1N0M0	Negative	Female	47	Yes	Patient alive with no evidence of disease 14+ years after diagnosis
***UM-SCC-105***	Larynx	T4N0M0	HPV18	Male	51	No	Patient alive with no evidence of disease 5+ years after diagnosis
UM-SCC-92	Tongue	T2N0M0	Negative	Female	38	No	Patient alive with no evidence of disease 4+ years after diagnosis
***UPCI:SCC152***	Recurrent Hypopharynx	T2N1M0	HPV16	Male	47	Yes	Patient died from disease 4 years after diagnosis

**Table 2:** Wes Antibody Information.

Antibody	Clone	Supplier	Catalog #	Lot	Secondary
Annexin V	N/A	Novus	NB100-1930	4E15L33570	Rabbit
βCatenin	D10AB	Cell Signaling	8480	5	Rabbit
Calnexin	N/A	Novus	NB100-1965ss	D-2	Rabbit
CD59	N/A	R & D Systems	AF1987	KIF011911A	Goat
CD9	C-4	Santa Cruz	SC-13118	E1719	Mouse
Cyclin D1	H-295	Santa Cruz	sc-753	G2315	Rabbit
Cytokeratin 19	A53-B/A2	Santa Cruz	SC6278	D1618	Mouse
E-Cadherin	180215	R & D Systems	MAB1838	JAT0219051	Mouse
EGFR	N/A	Origene	TA312545	20100915076	Rabbit
EPHA2	C-3	Santa Cruz	SC-398832	D2519	Mouse
HLA-A	EP1395Y	Abcam	ab52922	GR25873	Rabbit
HPV16 E7	N/A	Santa Cruz	SC-65711	I1216	Mouse
p16	N/A	Ventana	725-4793	E04025	Mouse
p53	DO-1	Neomarkers	MS-187-p	187p1201E	Mouse
Rb	1F8	Neomarkers	MS-107-p1	107p1607F	Mouse
STAT3	124H6	Cell Signaling	9139	7	Mouse
Tenascin-C	EPR4219	Abcam	ab108930	GR3209212-7	Rabbit

## Data Availability

The data supporting the figures presented in this study are openly available in FigShare at [https://figshare.com/s/3d3cd935872558e323f2, accessed on 25 June 2021]. Public DOI 10.6084/m9.figshare.14378672.

## Supplementary Materials

The following are available online at https://www.mdpi.com/article/10.3390/cancers13153714/s1, Table S1. Proteins from HNSCC cell line EVs with averaged fold-change peptide spectrum matches (PSM) >2 compared with the EVs from the normal keratinocyte line (NOKsi) (n = 149); Table S2.Wes protein intensity values for Figure 3; Table S3. Wes protein intensity values for Figure 8; Figure S1. CD9 and AnnexinV Wes protein quantitative luminosity for extracellular vesicles from HNSCC, normal keratinocyte, and transformed cell line; and Figure S2. All uncropped Wes gels showing protein bands and molecular weight markers.
