# Supplementary material for: Differences in Extracellular Vesicle Protein Cargo Are Dependent on Head and Neck Squamous Cell Carcinoma Cell of Origin and Human Papillomavirus Status
Source: Cancers (Basel). 2021 Jul 23;13(15):3714. doi: 10.3390/cancers13153714 (PMC8345072; doi:10.3390/cancers13153714)
Supplement: Supplementary file 1 [file cancers-13-03714-s001.zip › Figure S1.pdf]

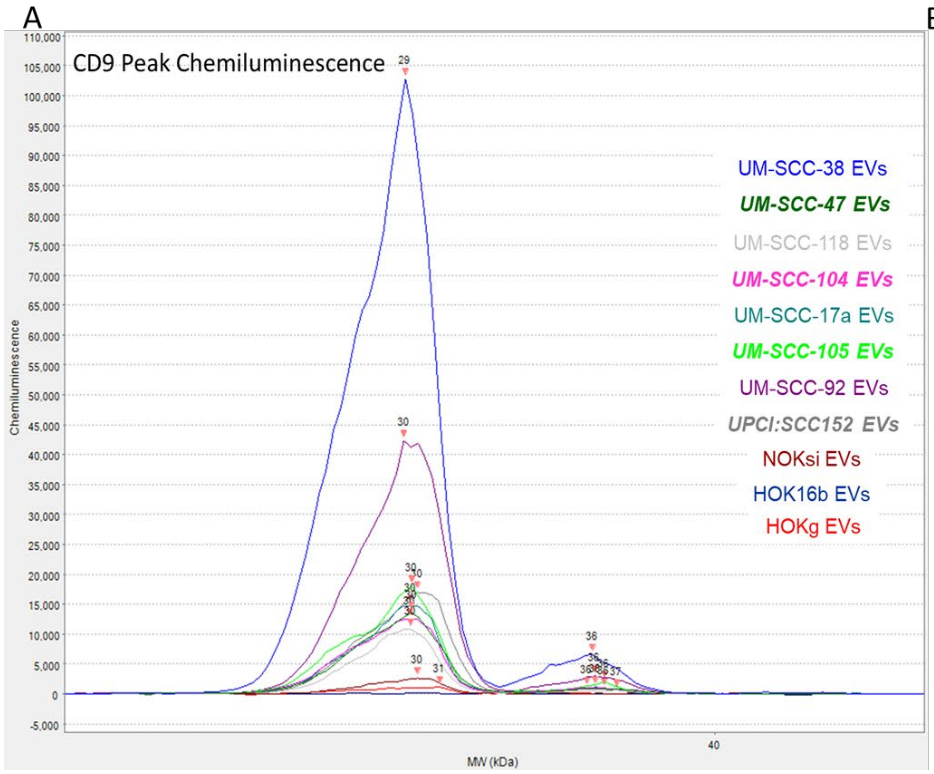

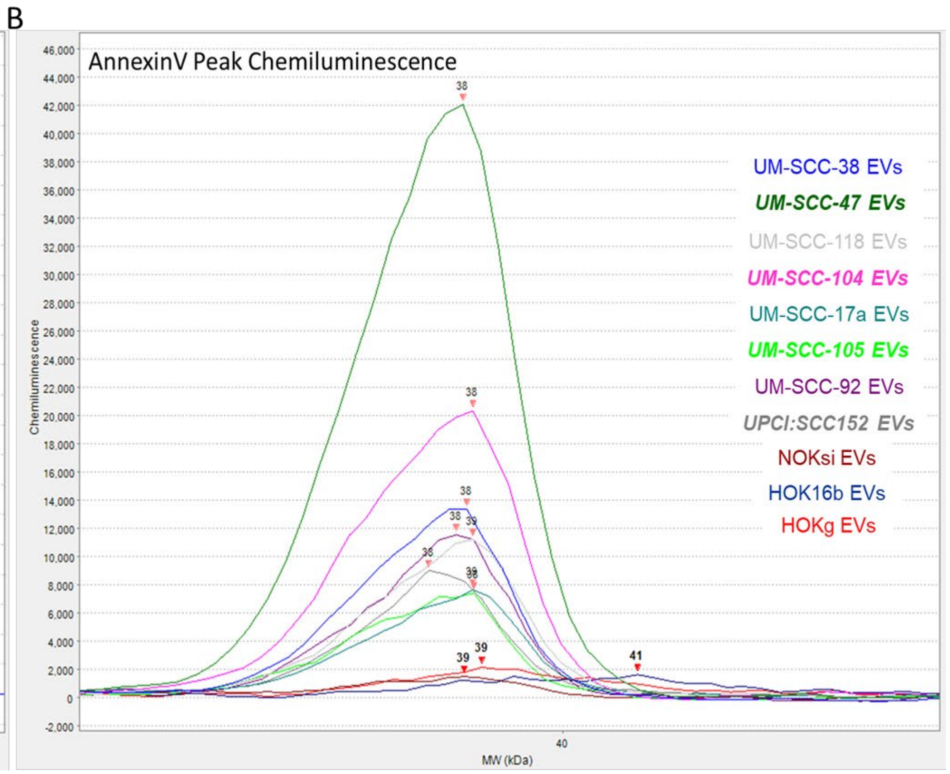

Figure S1. Wes protein quantitative luminosity for extracellular vesicles from HNSCC, normal keratinocyte, and transformed cell lines. (A) CD9 detected at 30kDa, 1:25 antibody dilution, 1μg/μL protein (B) AnnexinV detected at 38kDa, 1:200 antibody dilution, 0.25 μg/μL protein.
