# Supplementary material for: Differences in Extracellular Vesicle Protein Cargo Are Dependent on Head and Neck Squamous Cell Carcinoma Cell of Origin and Human Papillomavirus Status
Source: Cancers (Basel). 2021 Jul 23;13(15):3714. doi: 10.3390/cancers13153714 (PMC8345072; doi:10.3390/cancers13153714)
Supplement: Supplementary file 1 [file cancers-13-03714-s001.zip › Figure S2.pdf]

Figure S2. All uncropped Wes gels showing protein bands and molecular weight markers.

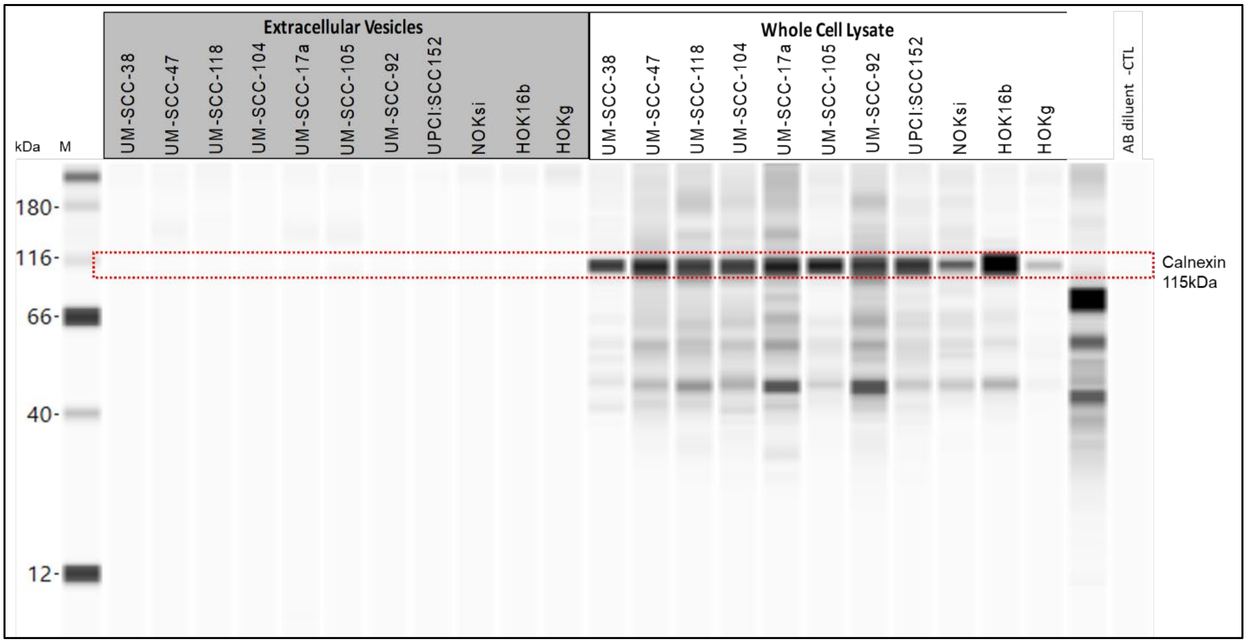

Panel A. Full Calnexin gel. Wes protein gel for extracellular vesicles and whole cell lysates from HNSCC, normal keratinocyte, and transformed cell lines. Calnexin detected at 115kDa, 1:50 antibody dilution, 1μg/μL protein.

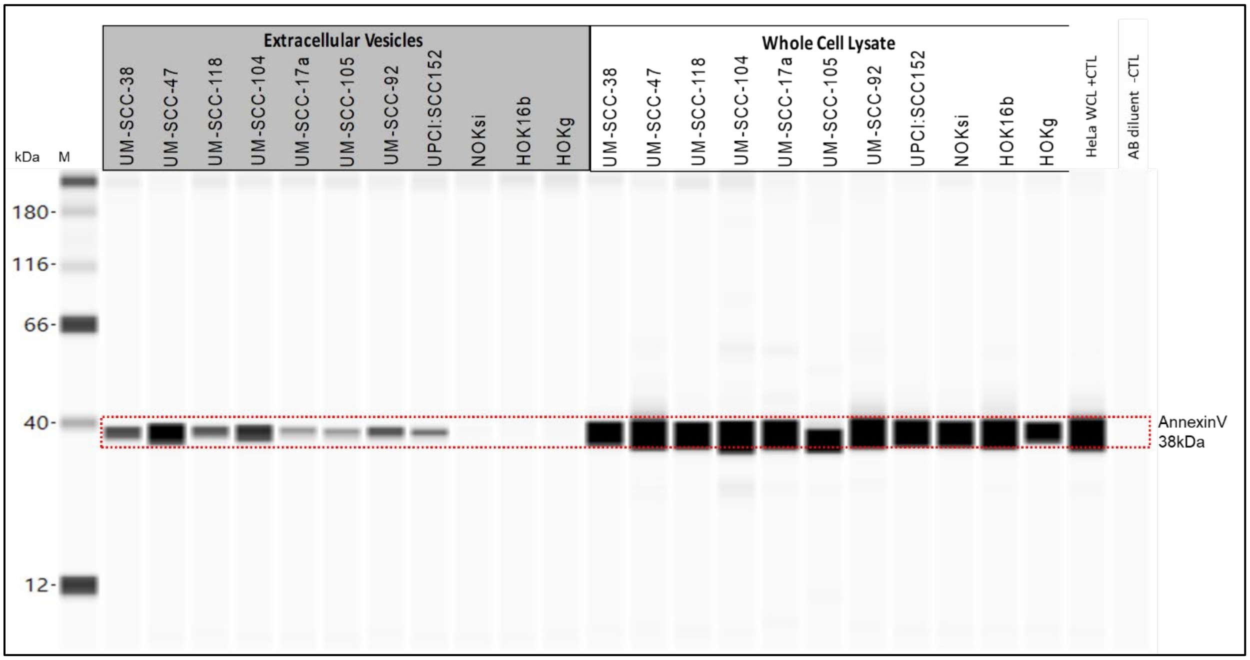

Panel B. Full Annexin Gel. Wes protein gel for extracellular vesicles and whole cell lysates from HNSCC, normal keratinocyte, and transformed cell lines. AnnexinV detected at 38kDa, 1:200 antibody dilution, 0.25μg/μL protein.

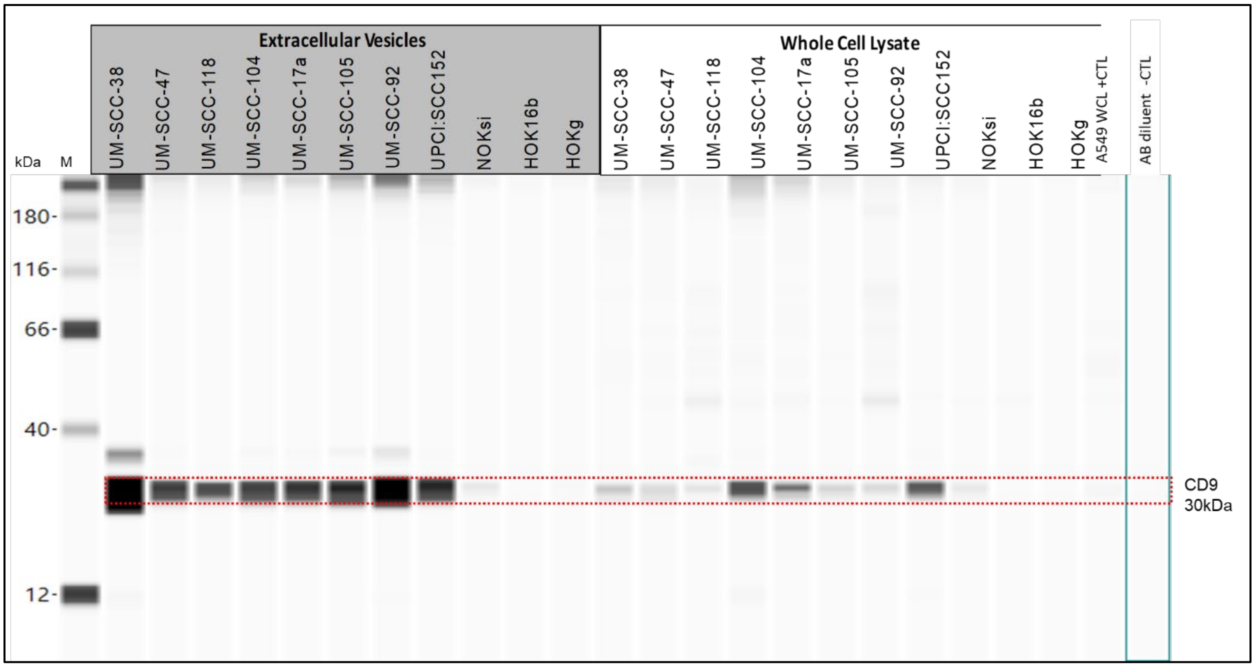

Panel C. Full CD9 gel. Wes protein gel for extracellular vesicles and whole cell lysates from HNSCC, normal keratinocyte, and transformed cell lines. CD9 detected at 30kDa, 1:25 antibody dilution, 1µg/µL protein.

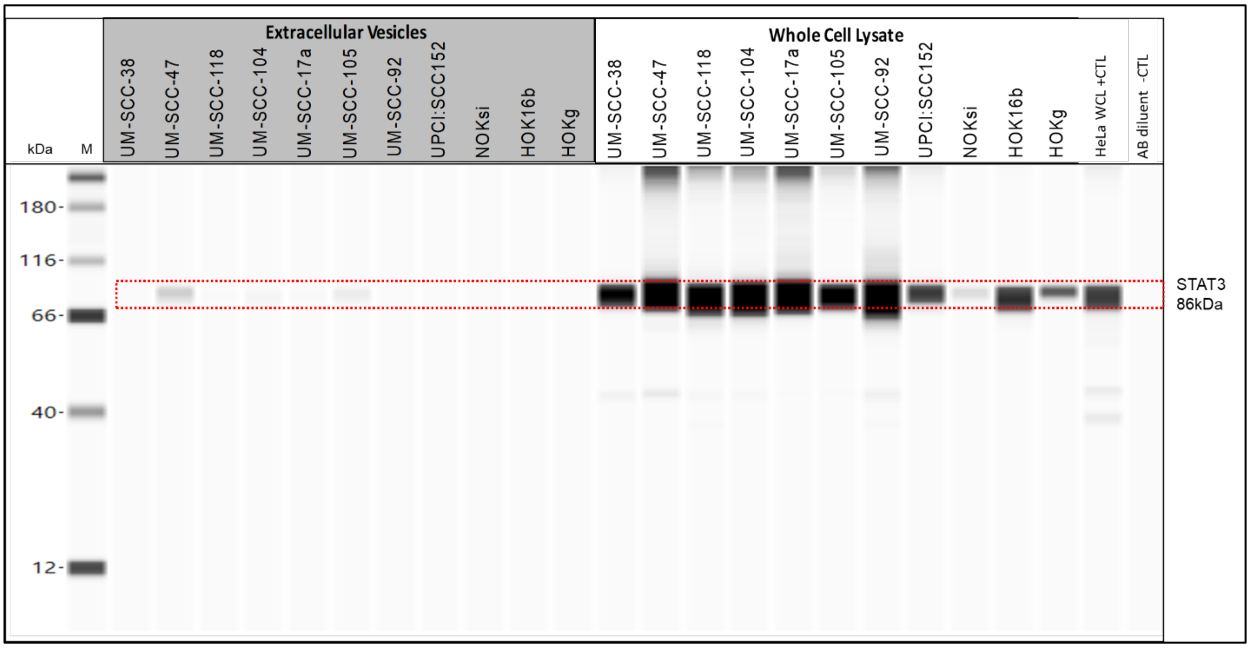

Panel D. Full STAT3 gel Wes protein gel for extracellular vesicles and whole cell lysates from HNSCC, normal keratinocyte, and transformed cell lines. STAT3 detected at 86kDa, 1:25 antibody dilution, 0.5µg/µL protein.

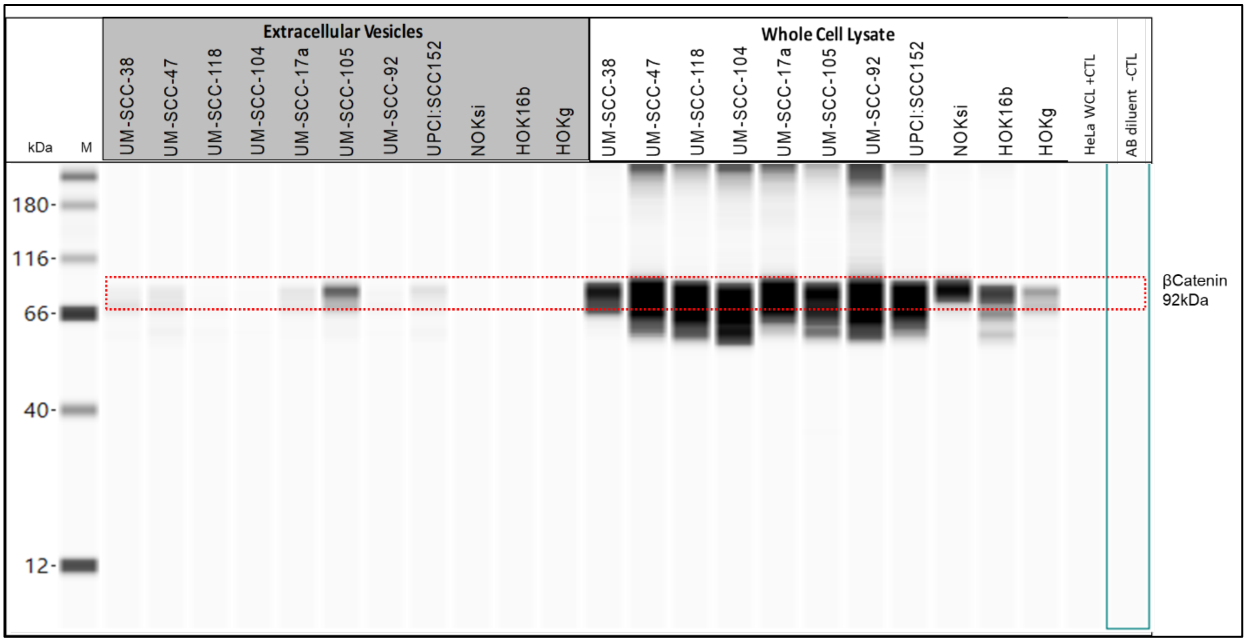

Panel E. Full Bcat gel. Wes protein gel for extracellular vesicles and whole cell lysates from HNSCC, normal keratinocyte, and transformed cell lines. βCatenin detected at 92kDa, 1:200 antibody dilution, 0.25µg/µL protein.

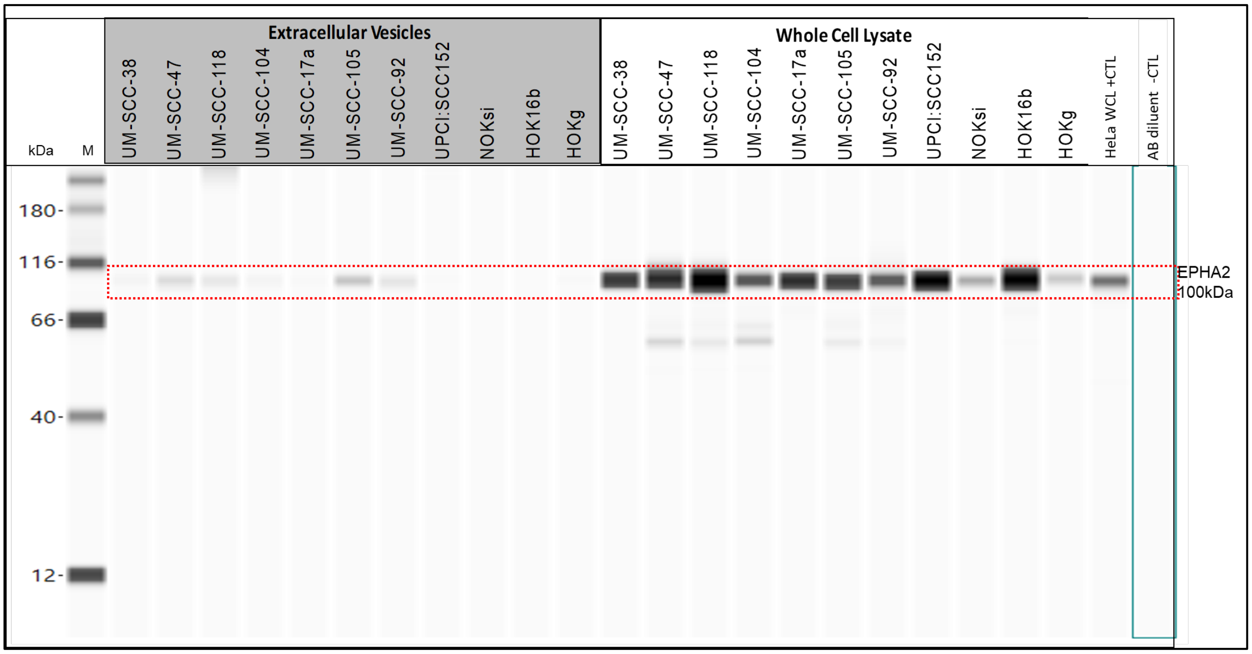

Panel F. Full EPHA2 gel. Wes protein gel for extracellular vesicles and whole cell lysates from HNSCC, normal keratinocyte, and transformed cell lines. EPHA2 detected at 100kDa,1:100 antibody dilution, 0.5mg/mL protein.

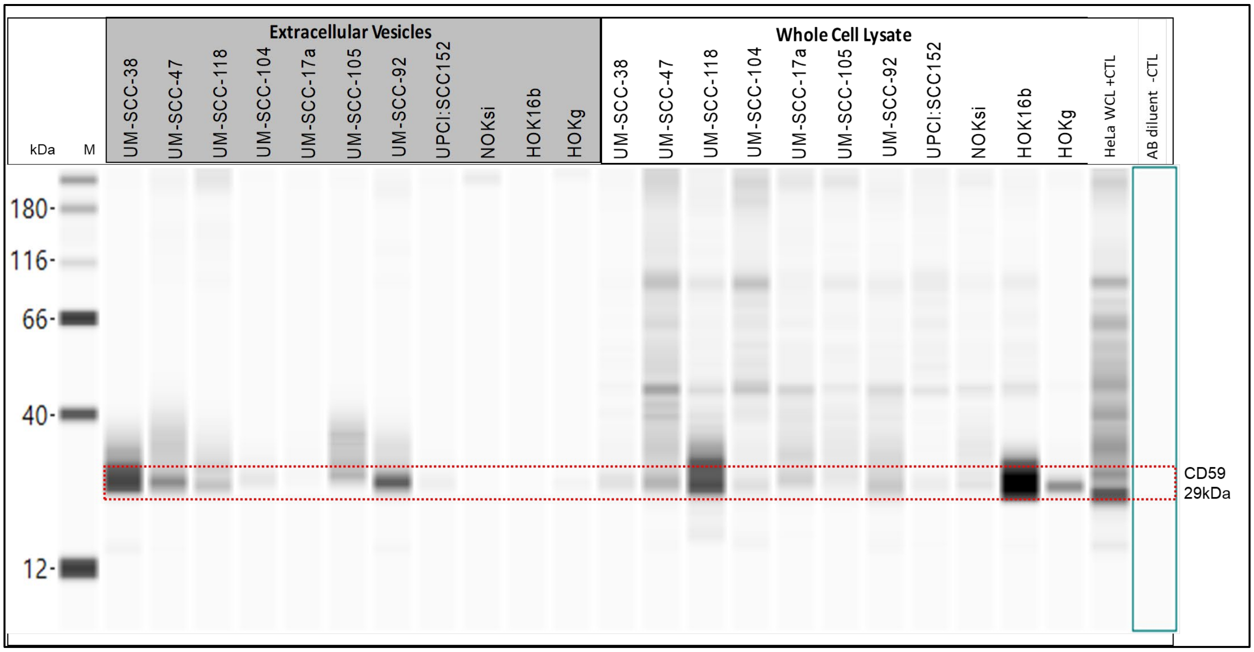

Panel G. Full CD59 gel. Wes protein gel for extracellular vesicles and whole cell lysates from HNSCC, normal keratinocyte, and transformed cell lines. CD59 detected at 29kDa, 1:7 antibody dilution, 1µg/µL protein.

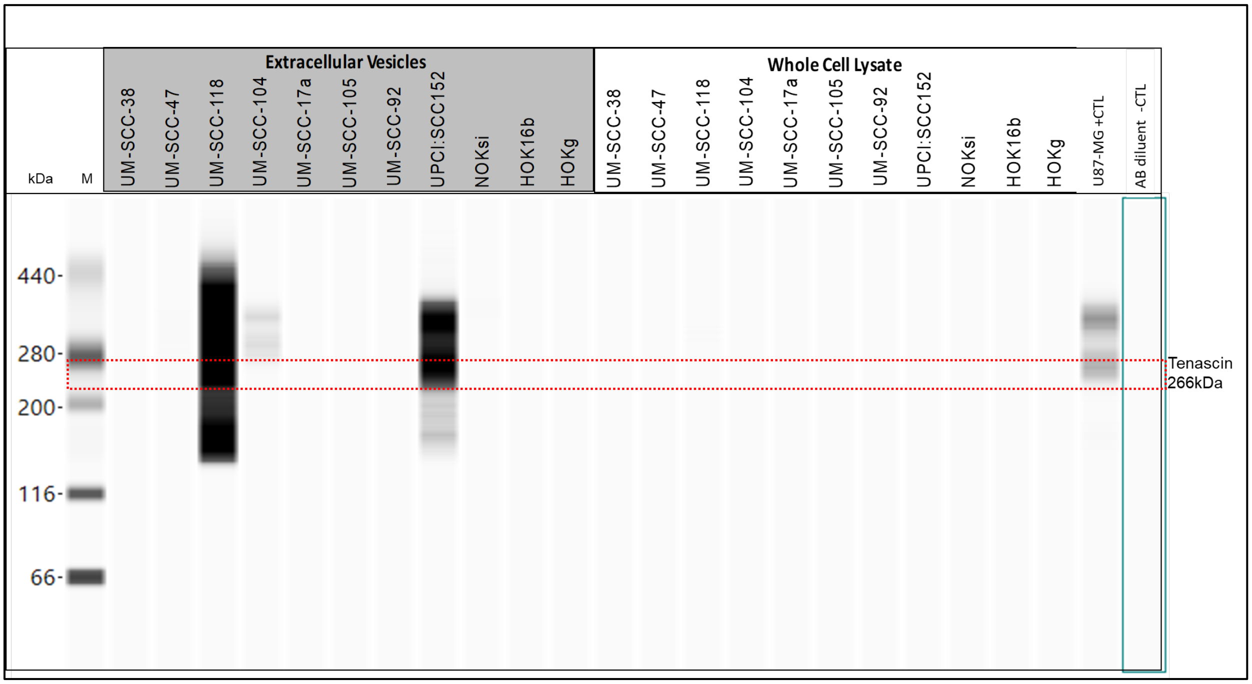

Panel H. Full TNC gel. Wes protein gel for extracellular vesicles and whole cell lysates from HNSCC, normal keratinocyte, and transformed cell lines. Tenascin detected at 266kDa, 1:200 antibody dilution, 0.5µg/µL protein.

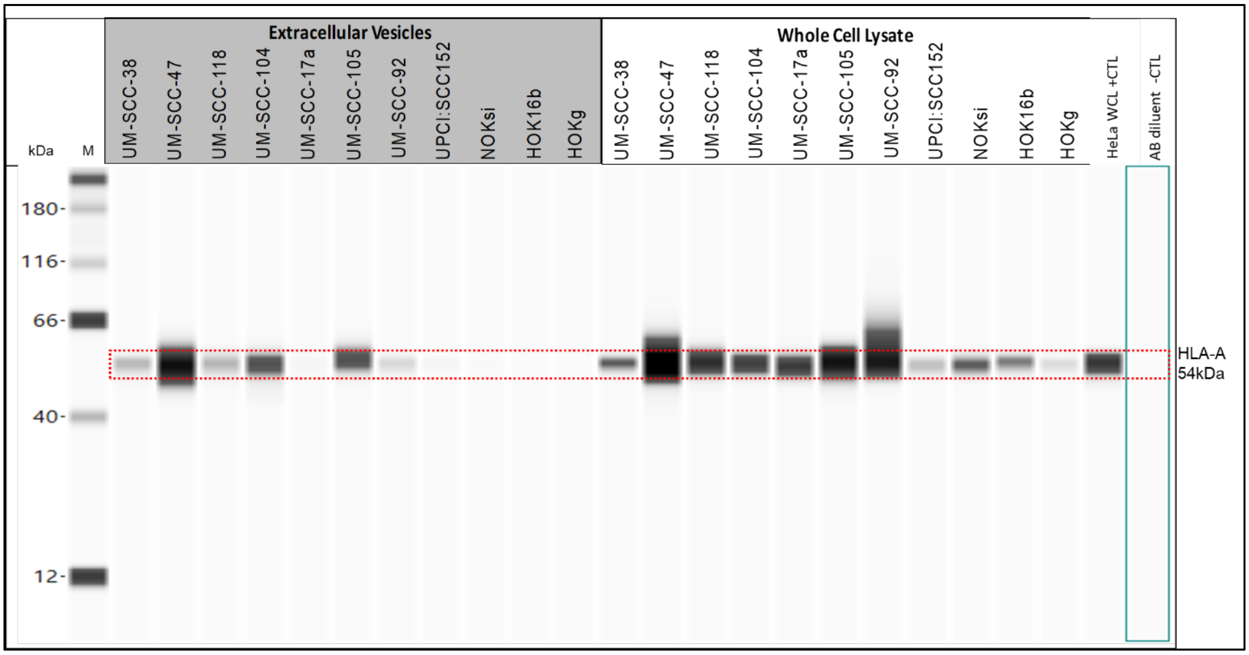

Panel I. Full HLA-A gel Wes protein gel for extracellular vesicles and whole cell lysates from HNSCC, normal keratinocyte, and transformed cell lines. HLA-A detected at 54kDa, 1:400 antibody dilution, 0.25µg/µL protein.

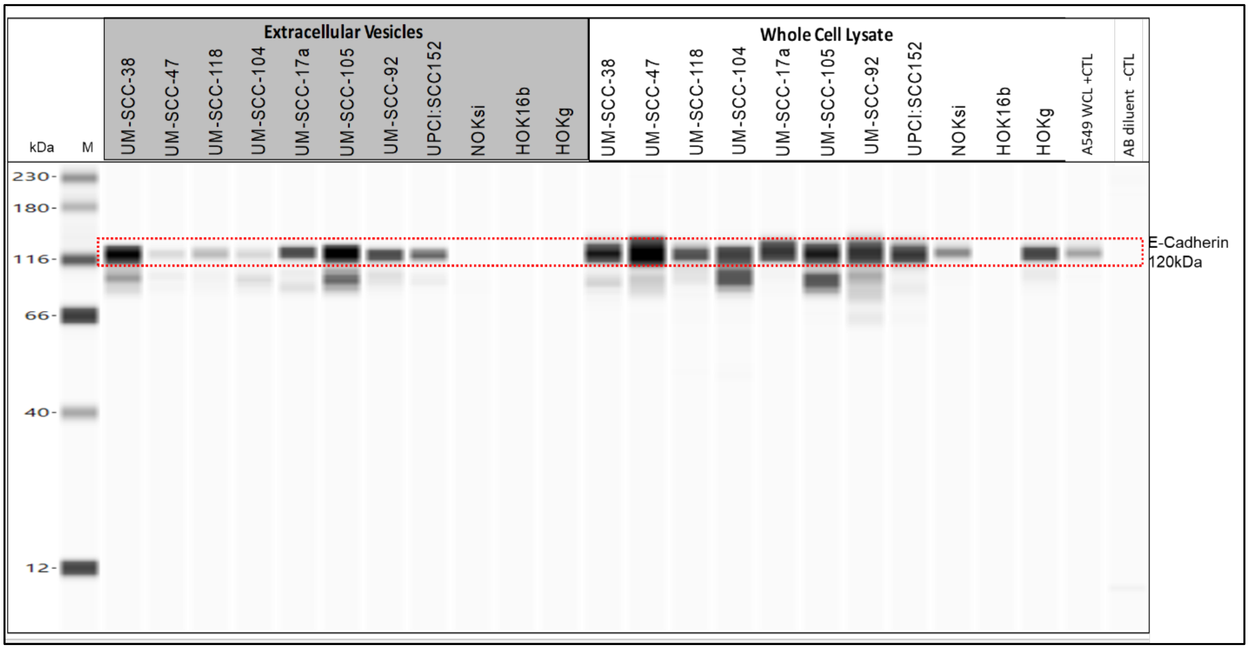

Panel J. Full Ecad gel Wes protein gel for extracellular vesicles and whole cell lysates from HNSCC, normal keratinocyte, and transformed cell lines. E-Cadherin detected at 120kDa, 1:250 antibody dilution, 0.5µg/µL protein.

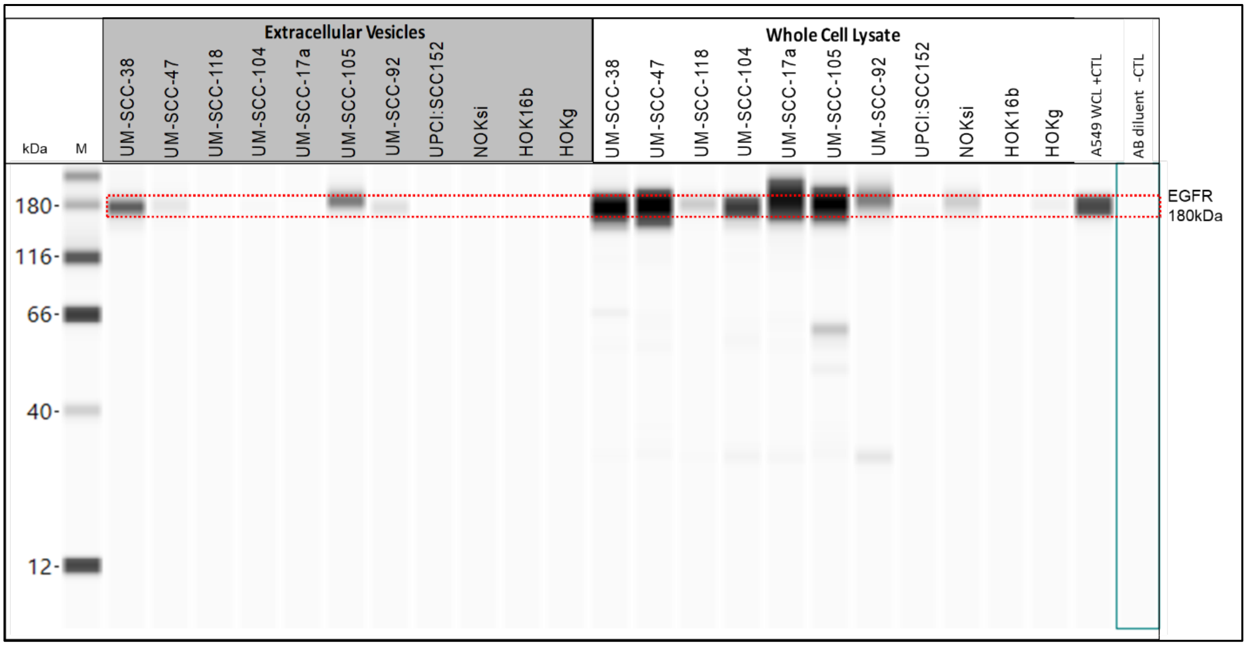

Panel K. Full EGFR gel. Wes protein gel for extracellular vesicles and whole cell lysates from HNSCC, normal keratinocyte, and transformed cell lines. EGFR detected at 180kDa 1:100 antibody dilution, 0.25µg/µL protein.

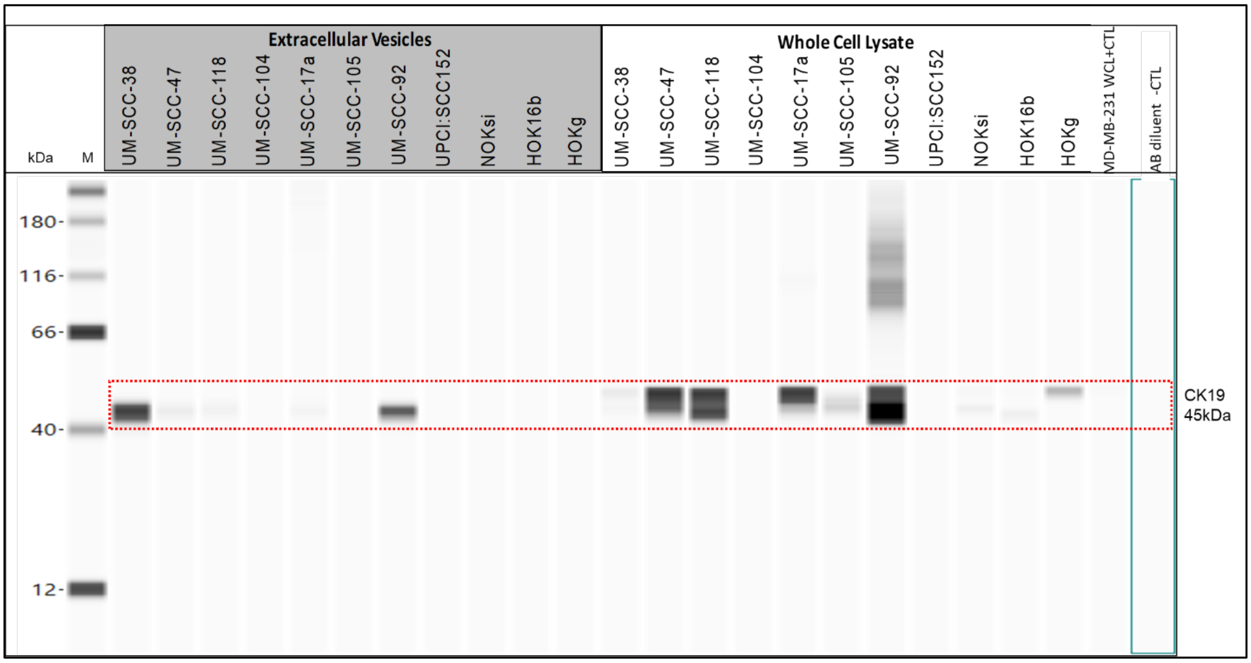

Panel L. Full CK19 gel. Wes protein gel for extracellular vesicles and whole cell lysates from HNSCC, normal keratinocyte, and transformed cell lines. CK19 detected at 45kDa, 1:100 antibody dilution, 0.25mg/mL protein.

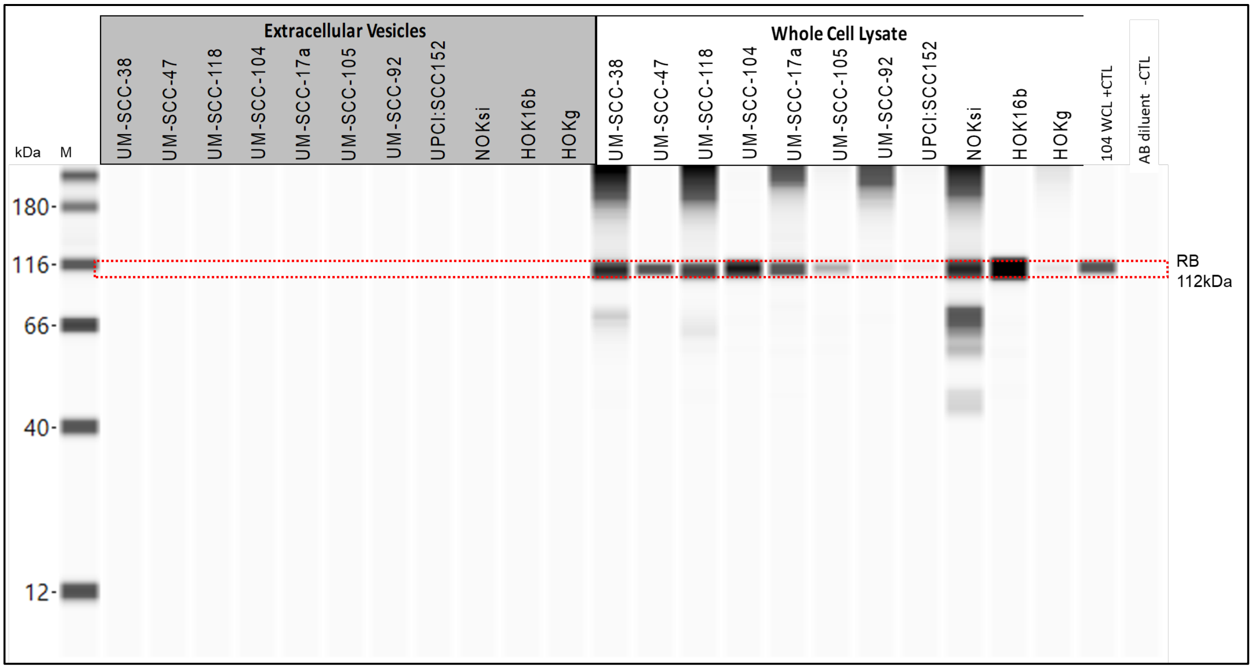

Panel M. Full RB gel. Wes protein gel for extracellular vesicles and whole cell lysates from HNSCC, normal keratinocyte, and transformed cell lines. RB detected at 112kDa, 1:100 antibody dilution, 0.5µg/µL protein.

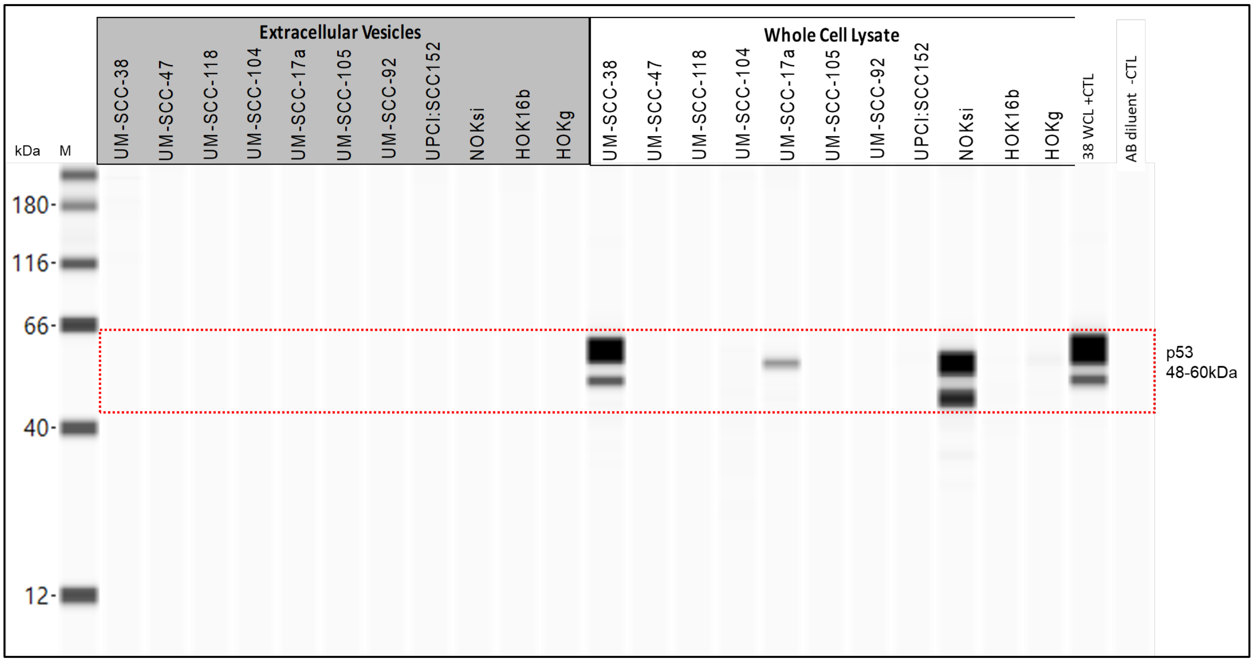

Panel N. Full p53 gel. Wes protein gel for extracellular vesicles and whole cell lysates from HNSCC, normal keratinocyte, and transformed cell lines. P53 detected at 60kDa, 52kda, and 48kDa, 1:50 antibody dilution, 0.25µg/µL protein.

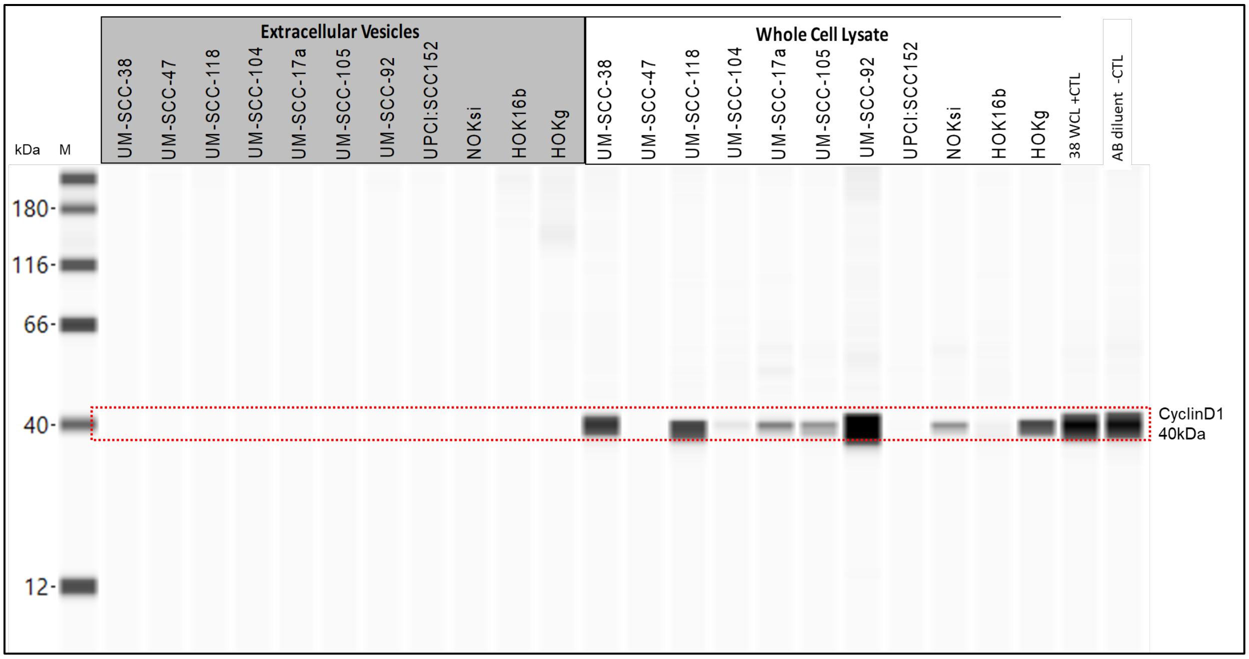

Panel O. Full Cyclin D1 gel. Wes protein gel for extracellular vesicles and whole cell lysates from HNSCC, normal keratinocyte, and transformed cell lines. CyclinD1 detected at 40kDa, 1:200 antibody dilution, 0.25µg/µL protein.

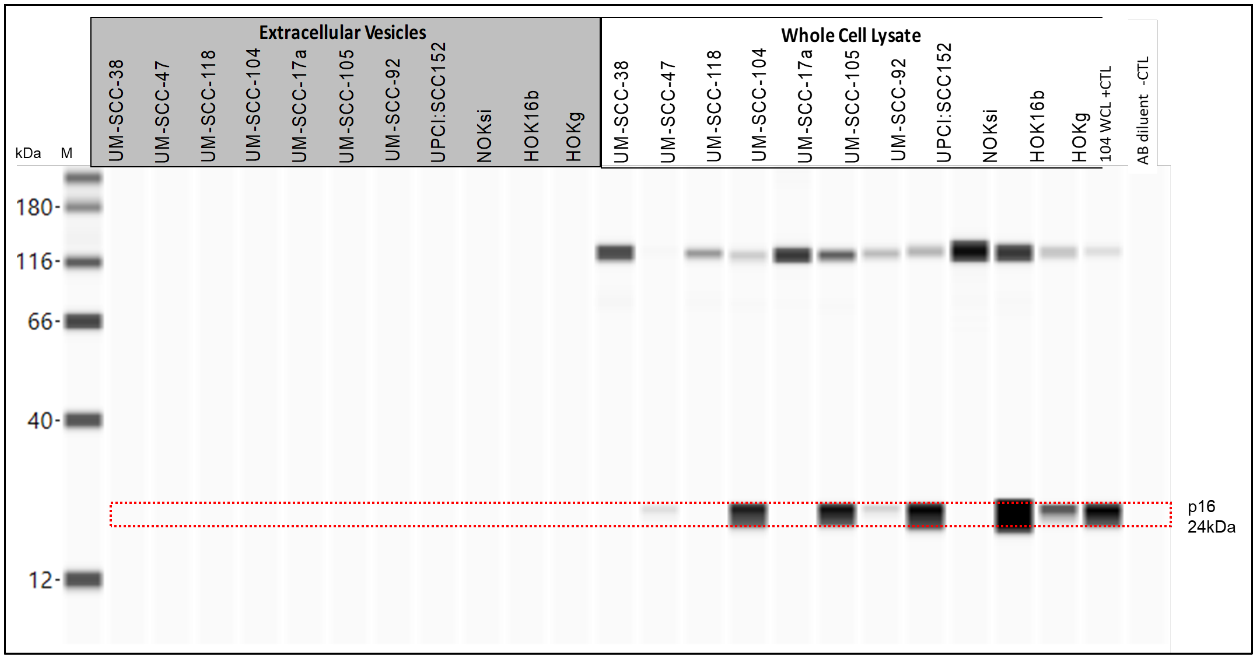

Panel P. Full p16 gel. Wes protein gel for extracellular vesicles and whole cell lysates from HNSCC, normal keratinocyte, and transformed cell lines. P16 detected at 24kDa, no antibody dilution, 0.25µg/µL protein.

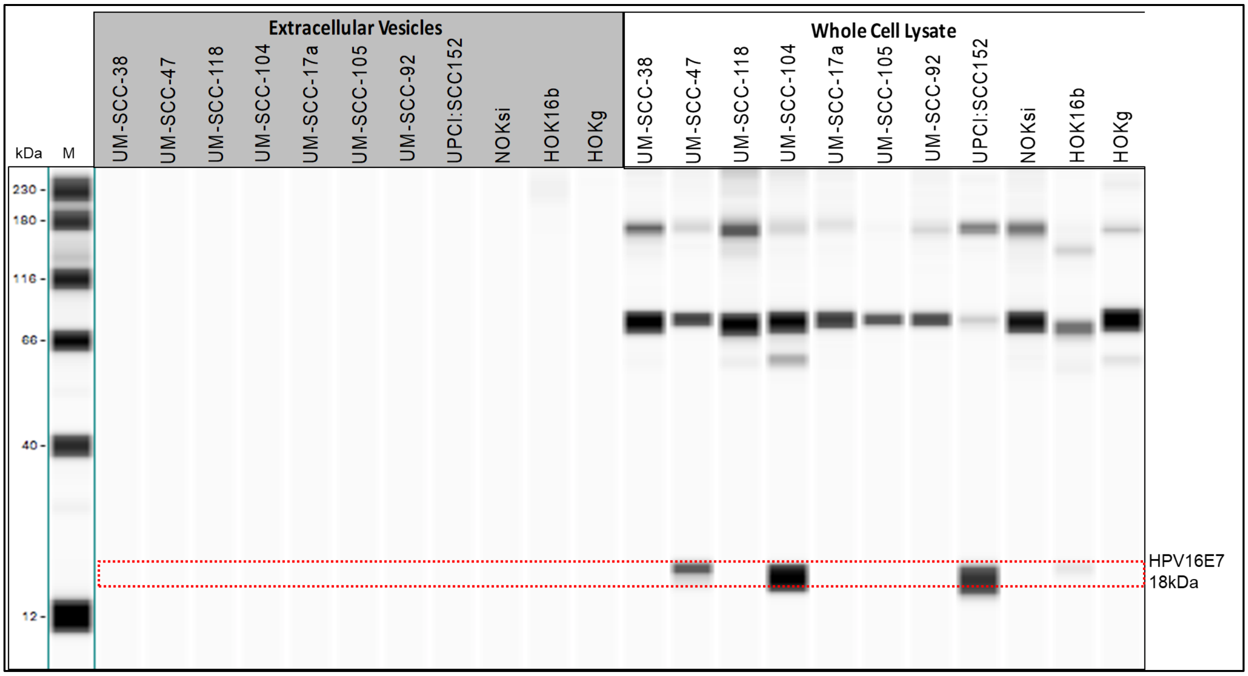

Panel Q. Full HPV16E7 gel. Wes protein gel for extracellular vesicles and whole cell lysates from HNSCC, normal keratinocyte, and transformed cell lines. HPV16E7 detected at 18kDa, 1:100 antibody dilution, 1µg/µL protein.
