# Supplementary material for: Differences in Extracellular Vesicle Protein Cargo Are Dependent on Head and Neck Squamous Cell Carcinoma Cell of Origin and Human Papillomavirus Status
Source: Cancers (Basel). 2021 Jul 23;13(15):3714. doi: 10.3390/cancers13153714 (PMC8345072; doi:10.3390/cancers13153714)
Supplement: Supplementary file 1 [file cancers-13-03714-s001.zip › Table S1.pdf]

Table S1. Proteins from HNSCC cell line EVs with averaged fold-change peptide spectrum matches (PSM) >2 compared to the EVs from the normal keratinocyte line (NOKsi) (n=149)

Accession ID	Protein
O75508	Claudin-11
Q9NRW3	DNA dC->dU-editing enzyme APOBEC-3C
A0A1B0GUN9	Espin
Q6UXH8	Collagen and calcium-binding EGF domain-containing protein 1
O15446	DNA-directed RNA polymerase I subunit RPA34
Q9H7N4	Splicing factor, arginine/serine-rich 19
Q96QC0	Serine/threonine-protein phosphatase 1 regulatory subunit 10
O15523	ATP-dependent RNA helicase DDX3Y
O75781	Paralemmin-1
O15551	Claudin-3
E9PDY6	Mucin-4
O60711	Leupaxin
Q9HCE6	Rho guanine nucleotide exchange factor 10-like protein
F5H550	C-type lectin domain family 18 member C
A0A0A6YYL4	Coronin
P15169	Carboxypeptidase N catalytic chain
A0A1W2PRA1	Stromal interaction molecule 2
Q8N142	Adenylosuccinate synthetase isozyme 1
Q9H8M2	Bromodomain-containing protein 9
P02765	Alpha-2-HS-glycoprotein
P82932	28S ribosomal protein S6, mitochondrial
Q9ULT0	Tetratricopeptide repeat protein 7A
P36955	Pigment epithelium-derived factor
J3KMX3	Alpha-fetoprotein
Q9H4B7	Tubulin beta-1 chain
P01023	Alpha-2-macroglobulin
P02042	Hemoglobin subunit delta
P20742	Pregnancy zone protein
P04745	Alpha-amylase 1
E9PGA6	C1QTNF3-AMACR readthrough (NMD candidate)
P51884	Lumican
P10620	Microsomal glutathione S-transferase 1
Q06033	Inter-alpha-trypsin inhibitor heavy chain H3
Q92743	Serine protease HTRA1
Q96DE0	U8 snoRNA-decapping enzyme
P08263	Glutathione S-transferase A1
O60636	Tetraspanin-2
Q6PCB0	von Willebrand factor A domain-containing protein 1

P39059	Collagen alpha-1(XV) chain
P42771	Cyclin-dependent kinase inhibitor 2A
P36021	Monocarboxylate transporter 8
P16219	Short-chain specific acyl-CoA dehydrogenase, mitochondrial
Q14210	Lymphocyte antigen 6D
G3V2W1	Protein Z-dependent protease inhibitor
A0A0A0MRJ7	Coagulation factor V
P02538	Keratin, type II cytoskeletal 6A
O95183	Vesicle-associated membrane protein 5
P00734	Prothrombin
Q8WWB7	Glycosylated lysosomal membrane protein
Q6UXB3	Ly6/PLAUR domain-containing protein 2
Q8NFJ5	Retinoic acid-induced protein 3
P04004	Vitronectin
A0A0C4DFN2	Glucokinase (Hexokinase 4) regulator
P01266	Thyroglobulin
Q8WXI7	Mucin-16
Q14520	Hyaluronan-binding protein 2
P18463	HLA class I histocompatibility antigen, B-37 alpha chain
A4D126	D-ribitol-5-phosphate cytidylyltransferase
P05543	Thyroxine-binding globulin
Q7RTV2	Glutathione S-transferase A5
E7ESC7	Macrophage erythroblast attacher
P00488	Coagulation factor XIII A chain
P62318	Small nuclear ribonucleoprotein Sm D3
Q15847	Adipogenesis regulatory factor
P00742	Coagulation factor X
P19971	Thymidine phosphorylase
O00339	Matrilin-2
A0A087WXL6	Vacuolar protein sorting 11 (Yeast), isoform CRA_a
O75340	Programmed cell death protein 6
Q9BZD6	Transmembrane gamma-carboxyglutamic acid protein 4
Q9P0J7	E3 ubiquitin-protein ligase KCMF1
Q8NFL0	UDP-GlcNAc:betaGal beta-1,3-N-acetylglucosaminyltransferase 7
Q14117	Dihydropyrimidinase
P13647	Keratin, type II cytoskeletal 5
Q6V0I7	Protocadherin Fat 4
P04114	Apolipoprotein B-100
B7ZL91	Meprin A subunit
Q9GZN4	Brain-specific serine protease 4
A8K0G1	Protein Wnt

O95873	Uncharacterized protein C6orf47
Q9HB40	Retinoid-inducible serine carboxypeptidase
P35625	Metalloproteinase inhibitor 3
O15393	Transmembrane protease serine 2
Q7Z406	Myosin-14
P01024	Complement C3
F8WAL6	Abl interactor 2
Q8N271	Prominin-2
P35321	Cornifin-A
Q96PE2	Rho guanine nucleotide exchange factor 17
P35443	Thrombospondin-4
P09758	Tumor-associated calcium signal transducer 2
Q5UIP0	Telomere-associated protein RIF1
Q75QN2	Integrator complex subunit 8
P20908	Collagen alpha-1(V) chain
Q04695	Keratin, type I cytoskeletal 17
P43121	Cell surface glycoprotein MUC18
P98160	Basement membrane-specific heparan sulfate proteoglycan core protein
O95832	Claudin-1
P02533	Keratin, type I cytoskeletal 14
Q17RR3	Pancreatic lipase-related protein 3
P10643	Complement component C7
P08727	Keratin, type I cytoskeletal 19
P05362	Intercellular adhesion molecule 1
Q8IYM9	E3 ubiquitin-protein ligase TRIM22
P05187	Alkaline phosphatase, placental type
O43653	Prostate stem cell antigen
Q76M96	Coiled-coil domain-containing protein 80
P05787	Keratin, type II cytoskeletal 8
Q96N76	Urocanate hydratase
P47929	Galectin-7
G3V1A6	Gasdermin domain containing 1, isoform CRA_d
Q9NRL3	Striatin-4
P29317	Ephrin type-A receptor 2
Q9Y4P3	Transducin beta-like protein 2
Q8WWY8	Lipase member H
Q68BL7	Olfactomedin-like protein 2A
B0YIW2	Apolipoprotein C-III variant 1
P01891	HLA class I histocompatibility antigen, A-68 alpha chain
P09668	Pro-cathepsin H
Q9Y337	Kallikrein-5

Q9UBP6	tRNA (guanine-N(7)-)-methyltransferase
Q9BSE2	Transmembrane protein 79
Q9BUF5	Tubulin beta-6 chain
A1A4S6	Rho GTPase-activating protein 10
Q5JPI3	Uncharacterized protein C3orf38
Q8NI27	THO complex subunit 2
Q14678	KN motif and ankyrin repeat domain-containing protein 1
P19823	Inter-alpha-trypsin inhibitor heavy chain H2
O00469	Procollagen-lysine,2-oxoglutarate 5-dioxygenase 2
Q99715	Collagen alpha-1(XII) chain
P63172	Dynein light chain Tctex-type 1
Q9Y4K0	Lysyl oxidase homolog 2
Q3KQV9	UDP-N-acetylhexosamine pyrophosphorylase-like protein 1
Q9GZT5	Protein Wnt-10a
Q9Y478	5'-AMP-activated protein kinase subunit beta-1
P13646	Keratin, type I cytoskeletal 13
O15232	Matrilin-3
O15347	High mobility group protein B3
Q6PJI9	GATOR complex protein WDR59
P18510	Interleukin-1 receptor antagonist protein
Q5BKZ1	DBIRD complex subunit ZNF326
A0A0J9YXF2	Paraoxonase 2, isoform CRA_a
P29508	Serpin B3
Q14573	Inositol 1,4,5-trisphosphate receptor type 3
P27482	Calmodulin-like protein 3
Q9UHV7	Mediator of RNA polymerase II transcription subunit 13
Q12884	Prolyl endopeptidase FAP
Q9HB63	Netrin-4
