# Supplementary material for: Differences in Extracellular Vesicle Protein Cargo Are Dependent on Head and Neck Squamous Cell Carcinoma Cell of Origin and Human Papillomavirus Status
Source: Cancers (Basel). 2021 Jul 23;13(15):3714. doi: 10.3390/cancers13153714 (PMC8345072; doi:10.3390/cancers13153714)
Supplement: Supplementary file 1 [file cancers-13-03714-s001.zip › Table S2.pdf]

Table S2. Wes protein intensity values for Figure 3. Bold italics: HPV-positive.

**Sample**	**Average Protein Intensity (Luminescence)**		
	**AnnexinV**	**Calnexin**	**CD9**
UM-SCC-38 EV	37549.0	0.0	271208.4
UM-SCC-47 EV	110471.0	0.0	17804.1
UM-SCC-118 EV	26844.3	0.0	44270.8
UM-SCC-104 EV	47951.1	0.0	40871.1
UM-SCC-17A EV	26217.7	0.0	132878.9
UM-SCC-105 EV	24363.8	0.0	133820.5
UM-SCC-92 EV	50674.7	0.0	42688.4
UPCISCC:152 EV	29584.3	0.0	137184.8
NOKsi EV	6837.7	0.0	13536.0
HOK16b EV	9839.3	0.0	13412.5
HOKg EV	5411.7	454.3	1147.1
UM-SCC-38 WCL	192031.6	59254.7	7835.5
UM-SCC-47 WCL	243144.6	26621.2	2833.6
UM-SCC-118 WCL	288892.6	38561.5	6252.5
UM-SCC-104 WCL	475389.2	18504.3	27982.8
UM-SCC-17A WCL	355077.5	28261.6	21663.3
UM-SCC-105 WCL	271650.0	41279.2	8378.6
UM-SCC-92 WCL	317918.0	15292.3	3220.7
UPCISCC:152 WCL	175005.0	55347.7	15616.3
NOKsi WCL	303760.3	29611.0	5811.2
HOK16b WCL	326741.6	174614.4	1394.2
HOKg WCL	221713.7	22730.3	11219.1
