# Supplementary material for: Differences in Extracellular Vesicle Protein Cargo Are Dependent on Head and Neck Squamous Cell Carcinoma Cell of Origin and Human Papillomavirus Status
Source: Cancers (Basel). 2021 Jul 23;13(15):3714. doi: 10.3390/cancers13153714 (PMC8345072; doi:10.3390/cancers13153714)
Supplement: Supplementary file 1 [file cancers-13-03714-s001.zip › Table S3.pdf]

Table S3. Wes protein intensity values for Figure 8. Bold italics: HPV-positive.

Sample	Average Protein Intensity (Luminescence)				
	RB	p53	Cyclin D1	p16	HPV16E7
UM-SCC-38 EV	0.0	0.0	0.0	0.0	0.0
UM-SCC-47 EV	0.0	0.0	0.0	0.0	0.0
UM-SCC-118 EV	0.0	0.0	0.0	0.0	0.0
UM-SCC-104 EV	0.0	0.0	0.0	0.0	0.0
UM-SCC-17A EV	0.0	0.0	0.0	0.0	0.0
UM-SCC-105 EV	0.0	0.0	0.0	170.8	0.0
UM-SCC-92 EV	0.0	0.0	0.0	0.0	0.0
UPCISCC:152 EV	0.0	0.0	0.0	155.5	0.0
NOKsi EV	0.0	0.0	0.0	0.0	0.0
HOK16b EV	0.0	0.0	0.0	0.0	0.0
HOKg EV	0.0	0.0	0.0	0.0	0.0
UM-SCC-38 WCL	57226.5	28991.9	19889.6	0.0	0.0
UM-SCC-47 WCL	36356.6	0.0	0.0	4258.6	4411.2
UM-SCC-118 WCL	41300.8	3058.8	17562.9	0.0	0.0
UM-SCC-104 WCL	69298.5	0.0	3431.0	23571.0	13757.2
UM-SCC-17A WCL	33284.1	24008.6	9012.2	0.0	0.0
UM-SCC-105 WCL	18610.4	0.0	8403.9	27884.8	0.0
UM-SCC-92 WCL	6964.8	0.0	86235.8	5178.9	0.0
UPCISCC:152 WCL	7838.9	0.0	0.0	29703.8	8416.9
NOKsi WCL	59121.6	181244.4	8326.7	264.0	138.8
HOK16b WCL	195370.8	0.0	1268.2	164660.3	1462.1
HOKg WCL	9075.3	5151.9	15222.3	11675.8	0.0
